# Template-Free Ultrafast Directed Self-Assembly Using Biaxial Toggled Magnetic Fields

**DOI:** 10.1021/acsnano.5c09450

**Published:** 2025-07-30

**Authors:** Guillermo Camacho, Juan de Vicente

**Affiliations:** F2N2Lab, Magnetic Soft Matter Group, Department of Applied Physics, 16741 Faculty of Sciences, University of Granada, C/Fuentenueva s/n, Granada 18071, Spain

**Keywords:** magnetorheological fluids, magnetic colloids, continuous magnetic fields, pulsed magnetic fields, toggled magnetic fields, directed self-assembly

## Abstract

Speeding up the directed self-assembly of functional nanomaterials is a rapidly advancing area of research. Traditional self-assembly methods can be slow and limited by kinetic barriers. In this study, we demonstrate that the process can be dramatically accelerated for magnetic colloids when biaxial toggled magnetic fields (BTFs) are used. In this field configuration, a transversal pulsed magnetic field is superimposed perpendicular to the primary toggled magnetic field, facilitating faster phase separation in a model magnetic colloid. This approach offers enhanced control over aggregation dynamics by adjusting the field’s frequency and intensity and does not require any physical templates. Beyond structure control, the aggregation kinetics can also be precisely tuned. Within the context of magnetic materials, this method enables the formation of diverse and tunable structures such as chains, columns, depercolated aggregates, and percolating bands. BTFs further promote the formation of highly crystalline domains, enhancing the properties of the resulting self-assembled materials. While this technique is specifically tailored for magnetic systems, its versatility makes it relevant for the design and fabrication of functional nanomaterials. The ability to tune aggregation kinetics and achieve a range of structures may be beneficial for applications in photonics, electronics, and biomedicine.

## Graphical abstract

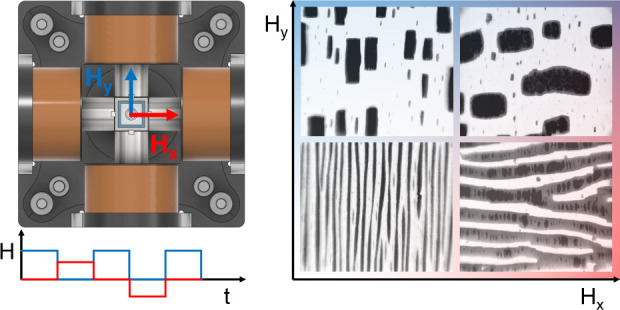

## 1 Introduction

Colloidal self-assembly constitutes a powerful strategy for creating complex, functional, and ordered structures that relies on the spontaneous organization of colloidal particles. This process is driven by intrinsic interactions among particles, between particles and the carrier medium, or with external boundaries, such as Brownian motion, gravity, van der Waals forces, or electrostatic interactions, among others. The ability to fine-tune particle interactions by modifying parameters like size, shape, surface chemistry, or confining geometry enables the development of functional materials with optical, mechanical, thermal, or chemical response (e.g., photonic crystals and metamaterials).^1–3^

Although spontaneous self-assembly has been widely studied, it encounters limitations such as kinetic barriers that hinder the system from reaching its minimum energy equilibrium state. As a result, suspensions become trapped in defective configurations, which obstructs crystallization.^4^ To overcome these kinetic traps, new pathways to equilibrium are required. In this context, directed self-assembly becomes relevant, defined by the application of external driving forces rather than relying exclusively on the intrinsic interactions within the system.^5^ Directed self-assembly involves the use of external stimuli, like electromagnetic fields or capillary, shear, acoustical and optical forces, temperature, pH and ionic strength to guide the assembly process.^6–8^ It offers a controlled approach to achieve optimal structures by modifying the particle interactions, which allows the design of hierarchically ordered structures^9–11^ or reconfigurable devices.^12,13^ In this study, we focus specifically on magnetic field-directed assembly.

Magnetic colloids (MCs) are typically formulated by dispersing Brownian magnetic particles in a nonmagnetic Newtonian liquid carrier.^14^ Under the sudden application of a sufficiently strong uniaxial time-independent (i.e., steady) external magnetic field, MCs form chain-like structures aligned along the field lines. However, when particle concentration is large enough, these structures percolate the sample volume^15^ resulting in lateral aggregation^16^ and the formation of loosely packed structures that are kinetically trapped in local energy minima.^17^ Interestingly, these field-induced structures determine the macroscopic behavior of the colloidal gel. In particular, a yield stress appears that can be externally controlled via the external magnetic field. This property has been extensively used in applications involving shock absorbers, dampers, and clutches.^18^ In addition, the field-aligned structures introduce a degree of anisotropy in the material with implications on macroscopic properties such as thermal conductivity^19^ and electrical or magnetic susceptibility.^20,21^

A versatile and efficient way to overcome kinetic barriers and the formation of percolating structures in far-from-equilibrium kinetically arrested states in MCs is the use of time-dependent fields instead of steady fields.^22,23^ The directed self-assembly of MCs under time-dependent magnetic fields is a rich and complex phenomenon that can be used to create novel materials with tunable structure and functionality.^11^ By adjusting the field configuration and the physicochemical properties of the MCs, one can control the formation of different ordered phases, offering insights into fundamental questions of phase behavior, kinetics, thermodynamics, and entropy in magnetic soft matter systems. One well-documented time-dependent magnetic field configuration is the case of sinusoidal fields. For example, high frequency biaxial rotatory fields invert the anisotropy of dipolar interactions, resulting in layered crystal structures.^24,25^ Moreover, precession fields generated by the superposition of a low frequency rotatory field with a perpendicular out-of-plane continuous field component enhance the collision of chain-like aggregates,^26^ promoting lateral aggregation, strengthening the structures and thereby improving their rheological response.^27^ Lastly, high frequency triaxial fields generated by the superposition sinusoidal fields along the three spatial axes suppress the dipolar interparticle interaction, leading to many-body interactions that result in isotropic network structures, or foams, with optimal physical properties.^21^

In addition to sinusoidal signals, uniaxial toggled (pulsed) fields (UTF), with a frequency that matches the reciprocal of the diffusion time of the particles, have also been used to coarsen the chain-like structures for short periods of time. These thicker structures exhibit a stronger rheological response (i.e., a larger yield stress) if compared to the structures formed under conventional steady magnetic fields.^28,29^ When the UTF is applied for a sufficiently long period of time, these coarsened chain-like structures collapse into low-energy, dense ellipsoidal aggregates.^30,31^ These dense aggregates exhibit an internal crystalline structure that strongly depends on the field frequency and the duty ratio (field on/off period ratio).^32^ This collapse into a crystalline structure results from the rearrangement of particles during field-off periods driven by Brownian motion,^33^ closely resembling a thermal annealing process.^34^ This justifies why depercolation of the structures only occurs within a narrow frequency range; if the toggling frequency is too low, particles diffuse a long distance during the field-off period and spatial correlations are lost in each cycle. If the toggling frequency is too high, particles are not capable to diffuse during the field-off period, and the structure remains kinetically arrested into chains.^35^

In this study, we propose the use of biaxial toggled fields (BTF) as a new route toward ultrafast self-assembly. The structuration process comprises the superposition of two alternative orthogonal UTFs, with one component serving as an externally tuned perturbation to the primary component in contrast to intrinsic Brownian motion (see Figure 1). The perturbation aims to hinder the repulsive region of the dipolar interaction and thus to promote lateral cluster aggregation. In classical steady fields, lateral aggregation of magnetic chains is severely limited by the repulsive nature of dipolar interactions in the perpendicular direction, and only occurs at later stages of assembly after initial particle chaining, with slow rates.^36^ This lateral aggregation of chains has been addressed by many authors, considering long-ranged^37^ and short-ranged interactions^38^ between chains. The former are dictated by Brownian motion while the latter are influenced by the chain length and both separation and displacement (in/out of registry) of the aggregates with respect to the field direction.^39^

**Figure 1:**
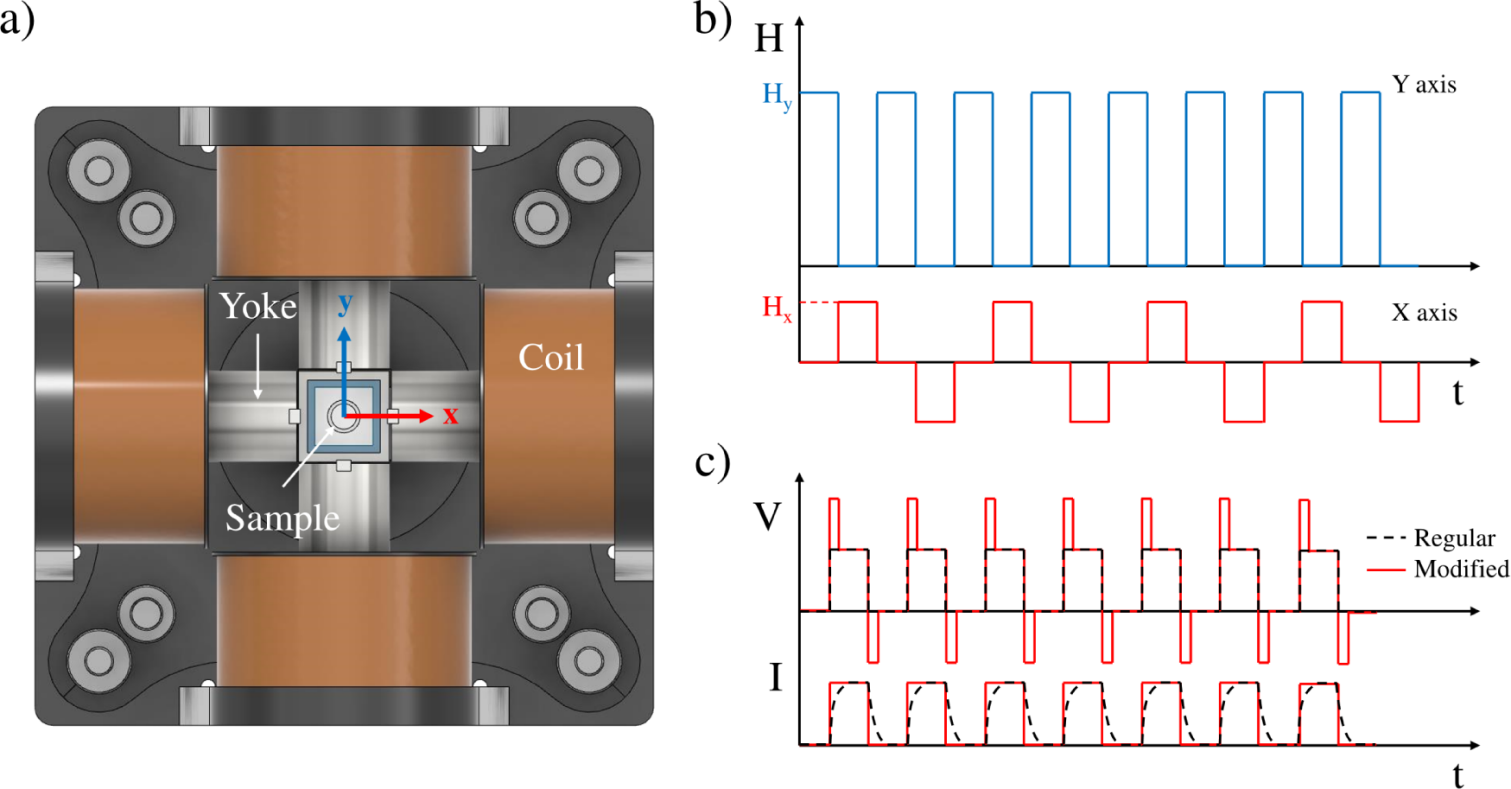
Schematics of the experimental device (a) and magnetic field configuration (b and c). (a) Two-dimensional slice of the triaxial magnetic field generator. (b) Biaxial toggled field (BTF) configuration: The Y axis is set as the primary axis and a perpendicular (perturbation) field in the X direction is applied in phase opposition. (c) Corrected pulse generation: To produce true square waves for the current (and consequently the field strength) at the higher frequencies examined in this study, a voltage overshoot is necessary (modified signal). Without this voltage adjustment, the current signal becomes deformed due to coil impedance (regular signal).

The use of perturbations to enhance self-assembly in MCs has been explored with different approaches. The use of multiaxial fields has been proposed as a tool for assembling colloids into crystals, chains, or sheets of tunable local symmetry [e.g.,^40–43^ ]. As an example, Donado and coworkers showed that the application of an orthogonal oscillating field to a steady field enhances structure dynamics, forming longer and thicker chains. The oscillating perturbation can make the chains oscillate around the steady field direction, thus keeping a preferred direction while favoring lateral aggregation.^44,45^ Similarly, the superposition of magnetic and electric fields has been shown by several authors to produce a tunable double-dipolar interaction^46,47^ that can form bidirectional chains or 2D clusters. Mechanical perturbations in the form of small oscillatory shear have also been used to promote lateral chain aggregation, improving physical properties such as conductivity.^48^

Our approach utilizing BTF involves superimposing toggled fields in phase opposition, offering precise control over both the field frequency and perturbation amplitude to balance the interaction between thermal diffusion and attractive forces. This BTF configuration is far more efficient than Brownian motion alone (uniaxial toggled fields) in forming larger aggregates in shorter times, while also promoting the formation of highly crystalline structures. This method provides enhanced control over the final microstructure, enabling the emergence of novel and exotic arrangements.

## 2 Results and Discussion

This section is organized into four main subsections. First, we describe the final self-assembled structures under BTFs, studying the influence of field frequency and relative strengths (Section 2.1). This allows us to construct a phase diagram using dimensionless parameters (Section 2.2). We then analyze the crystal structure of the assembled aggregates, comparing them to the steady field structuration (Section 2.3). Finally, we investigate the time evolution of the structures, paying special attention to the characteristic length of the aggregates and obtaining a master curve to explain the coarsening dynamics (Section 2.4).

### 2.1 Structure

MCs employed in this work were prepared from monodisperse magnetic latex particles (Dynabeads MyOne Carboxylic Acid, ThermoFisher). BTFs were applied using a triaxial field generator^49^ (see Figure 1) for at least 3000 s, allowing the system to reach a steady state during the mesoscopic coarsening process. Further details on field generation and sample preparation and characterization are provided in the Materials and Methods section. From this point forward, the *Y* field component (*H_y_*) will be considered the primary field, while the orthogonal toggled field along the *X* axis (*H_x_*) will be treated as a perturbation with a variable relative amplitude. In Figure 2 we show the structures formed at a particle surface fraction of ϕ_2D_ = 0.45 for three different field frequencies (*f* = 0.3, 1, and 3 Hz) as a function of the *Y* axis magnetic field strength (*H_y_*) and the field strength ratio (*H_x_*/*H_y_*) (i.e., the perturbation strength) in the range from 0.05 to 1. For all three frequencies investigated, uniformly distributed small-size aggregates are observed for low magnetic field strengths in both X and Y directions. However, heterogeneously distributed, large, and irregular, aggregates are observed for a higher field strength in any direction. Up to four different structures can be identified: Percolating chain-like and columnar structures along the primary field axis (*Y* axis), depercolated aggregates along the *Y* axis, depercolated aggregates along the orthogonal field axis (*X* axis) and percolating bands along the *X* axis.

**Figure 2:**
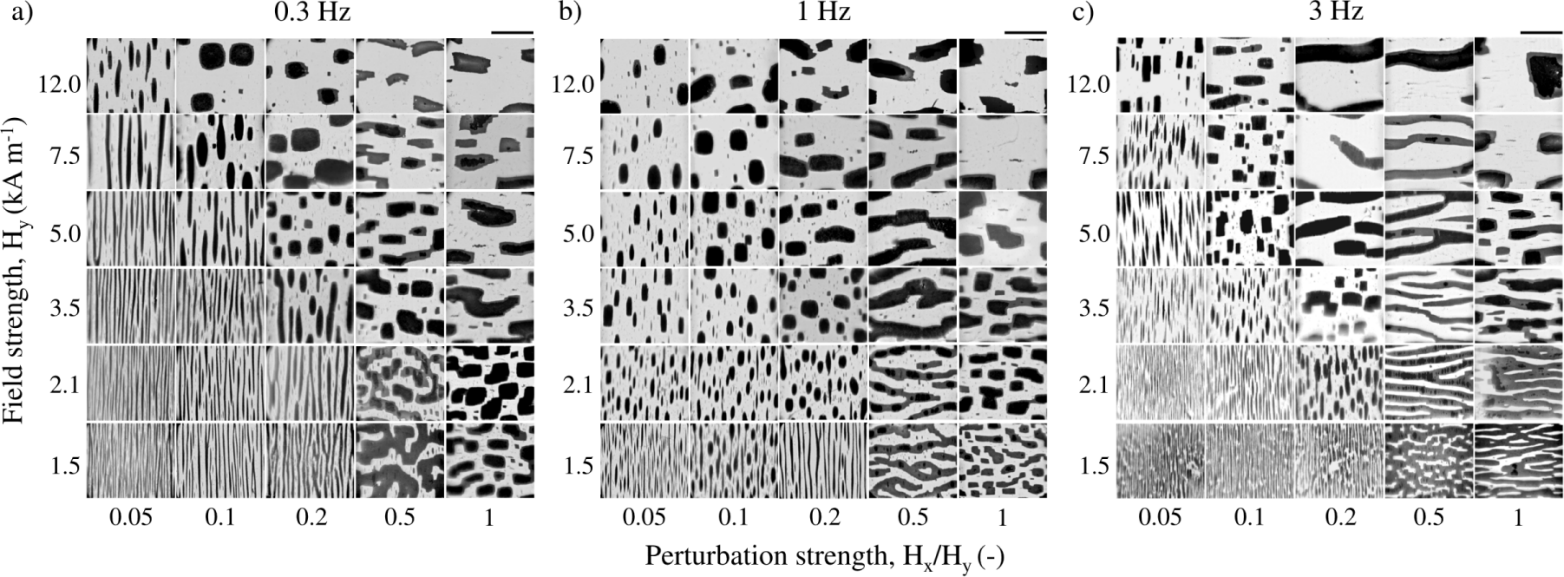
Micrographs of the structures formed after applying a sequence of BTFs for 3000 s with different frequencies, as a function of the primary field strength *H_y_* and perturbation strength *H_x_* /*H_y_*. (a) *f* = 0.3 Hz, (b) *f* = 1 Hz, and (c) *f* = 3 Hz. Differences in the transmitted light intensity through the aggregates also provide a useful indication of their thickness. Lighter areas correspond to the thickness of a single particle, while darker regions indicate the stacking of multiple layers. This stacking has been shown to be energetically favorable^31^ and also observed experimentally, forming a crystalline structure.^33^ The black scale bar in the top right corner indicates 500 μm.

As previously mentioned, the structures depicted in Figure 2 correspond to a particle surface fraction of ϕ_2D_ = 0.45. The influence of surface fraction on the resulting morphology is further examined in Supporting Information S1, where we show that, over long time scales, the aggregate morphology is highly dependent on particle concentration: low ϕ_2D_ values impede structural percolation, while higher values facilitate it. However, as will be shown in Section 2.4 and Supporting Information S5, the kinetics of structural evolution are unaffected by particle concentration, consistently displaying the same scaling behavior across varying ϕ_2D_ levels.

As a general rule, results obtained for the three different frequencies in the case of negligible perturbation fields (*H*_*x*_ → 0) closely resemble those reported in the previous literature for UTFs^33,35^ (see bottom left corners in Figure 2). Here, the perturbation field is weak enough for the thermal diffusion of the particles to dominate during the primary field-off periods. This explains the radically different structures found depending on the field frequency. Similar to the UTF, it is around *f* = 1 Hz, that structures collapse into ellipsoidal aggregates aligned with the primary field *H*_*y*_. Meanwhile, at *f* = 0.3 Hz and *f* = 3 Hz, the suspension remains arrested for longer times in columnar and chain-like structures, respectively [For a detailed discussion on the distinction between chains and columns, we refer the reader to Camacho et al. (2025)^35^]. This occurs because at 1 Hz, the field frequency matches the reciprocal of the typical diffusion time scale of the particles (*f* ∼ *t*_d_^–1^), defined by the time required for an isolated spherical particle to travel one diameter σ, due to thermal motion:^33^

1 $$t_{d}=\frac{3}{4}\frac{\pi \eta \sigma^{3}}{k_{B}T}$$

Here η is the carrier fluid viscosity, *k*_B_ is the Boltzmann constant and *T* (=298 K) is the absolute temperature. At this frequency (*f* = 1 Hz), the diffusion during a single period is sufficient to rearrange the structures without drastically altering them. This allows the structures to relax into highly ordered, compact ellipsoids, which have been shown to represent the energy minimum configuration for a UTF.^50^ In contrast, when the frequency is too low (0.3 Hz), the long thermal diffusion times lead to significant changes in the structure, erasing much of the spatial correlation between particles and preventing progressive relaxation into optimal configurations, resulting in the dominance of columnar aggregates. Finally, at higher frequencies (3 Hz), the period between successive pulses is short enough to hinder particle rearrangement via diffusive movement, kinetically trapping the particles in chain-like structures.

As the orthogonal perturbation field strength increases, new structures emerge. When the perturbation becomes significant, it dominates Brownian motion and the system is fully driven by the field. This causes aggregates to depercolate and coalesce into compact arrangements, even at frequencies far from the critical relaxation time scale. In the end, the interparticle interaction can be understood as a double-dipolar interaction in orthogonal directions, so that the repulsive regions of both polarizing fields are hindered. A uniaxial dipole–dipole interaction between two identical particles is attractive if the angle between the field vector and the line joining the dipole centers θ is below 54.7°:^14^

$${\mathrm{f⃗}}_{\mathrm{dip}}=-\frac{3{\mu_{c}m}^{2}}{4\pi r^{4}}\left[\left(3\cos^{2}\theta -1\right)\mathrm{r̂}+\sin\theta \mathrm{\theta ̂}\right]$$ 2

where *m* is the particle magnetic moment, μ_c_ is the permeability of the carrier and *r* the center-to-center interparticle distance. Klapp, Velev, and co-workers developed a double-dipolar model, obtained by superimposing orthogonal steady electric and magnetic fields which can be thought of as an analogue to our experimental system.^46^ By adjusting both field strengths, the degree of anisotropy was controlled, ultimately achieving fully attractive interactions. The authors also demonstrated that under certain field conditions, small-sized regions reconfigure into close-packed crystals. However, this crystallization occurs at a slow rate, with less than 0.12% of the particles forming compact arrangements after 1000 s of field superposition. This slow process is due to the steady fields used in this study, which trap the particles in local energy minima and prevent further relaxation from kinetically arrested states. In contrast, alternatively toggling the orthogonal fields imparts a much more dynamic character to the structures, allowing for continuous and long-lasting reconfiguration of the particles. This results in the formation of large, compact aggregates with high degrees of crystallinity in shorter time scales (see Section 2.4 for details on evolution dynamics). The use of toggled magnetic fields also eliminates the need for an additional dipolar interaction of a different nature, such as electric dipolar interactions.

In view of Figure 2, morphological differences arise as perturbation strength increases depending on field frequency. Higher frequencies restrict the available time for reconfiguration, and such a limited particle movement between successive periods results in quasi-rectangular aggregates with sharp edges. The lower frequencies, conversely, offer prolonged structuration times that destroy correlations between successive pulses and slow down aggregate coalescence.

It is worth noting the formation of thick band structures along the perturbation axis when the perturbation is of the order of the primary field component (*H*_*x*_ ∼ *H*_*y*_). These structures have been observed across various systems, including magnetotactic bacteria^51,52^ or suspensions affected by electrokinetic phenomena.^53–57^ Similar banded structures have recently gained attention and have been reported by^32^ under UTFs with a low duty ratio (*t*_on_ ≪ *t*_off_), where particle diffusion is significant relative to the structuring periods when an intense magnetic field is applied. However, BTFs offer a broader landscape for band formation and morphology, as the lateral diffusion can be controlled by the perturbation strength.

More recently, Kach and coworkers proposed a phenomenological model explaining this band formation through a modified dipolar force where the pair rotation component (second term in eq 2) can be adjusted.^58^ Although band formation in some systems has been attributed to hydrodynamic interactions,^59–61^ dipolar interactions with hindered pair rotation have been shown to sufficiently explain this structuration, even reproducing dynamic properties.^62^ The angular component of the dipolar interaction causes particles to align in the field direction, facilitating chain formation. In BTFs, both orthogonal fields generate opposite rotation components that cancel each other out, triggering band formation. These structures are highly dynamic, with particles continually circulating around the boundaries of each band (see Section 2.4.2 and Figure 9).

A rough estimation of the hindered pair rotation component can be obtained by computing the average interaction force through superposition of interaction fields from two orthogonal magnetic fields with relative strengths *H*_x_/*H*_y_. In the dipolar regime, neglecting mutual interaction, the modified biaxial interaction can be expressed as

$${\mathrm{f⃗}}_{\mathrm{BTF}}=-\frac{3\mu_{c}m^{2}}{4\pi r^{4}}\left\{\mathrm{r̂}\left[\left(3\cos^{2}\theta -1\right)+{\left(\frac{H_{x}}{H_{y}}\right)}^{2}\left(3\sin^{2}\theta -1\right)\right]+\left[1-{\left(\frac{H_{x}}{H_{y}}\right)}^{2}\right]\sin\left(2\theta \right)\mathrm{\theta ̂}\right\}$$ 3

where *m_y_* represents the magnetization along the primary field direction and θ is also measured in reference to this axis. This expression enables comparison with the rotational tuning parameter proposed by Kach and coworkers, as $\alpha =1-{\left(\frac{H_{x}}{H_{y}}\right)}^{2}$ . According to Kach et al. (2024)^58^, band formation occurs for α ≤ 0.4. In our experiments, however, we observe bands forming over a broader range, λ_*x*_/λ_*y*_ = [0.25–1] which holds across all frequencies examined in this study, corresponding to α =[0–0.75]. This discrepancy is likely due to the approximate nature of the averaged interaction, which is more accurate in the high-frequency limit, and does not capture the full dynamics of particle interactions.

Importantly, our system differs from Kach’s in another fundamental way: in addition to modifying the rotational component, the orthogonal field also alters the radial component of the interaction in a coupled manner. As shown in eq 3, the radial force is not invariant and its angular dependence changes with the field ratio. This leads to a reduction of the lateral repulsion present in classical dipolar systems, especially at high perturbation strengths. To illustrate this, in the Supporting Information S2 we include vector plots of the averaged interaction field for different perturbation strengths, highlighting both radial and rotational components. As the perturbation increases, the lateral repulsion vanishes, promoting the coalescence of particles into bands. In summary, while the hindered rotation framework offers a valuable analogy, our system exhibits richer physics due to the interplay of field components. The estimation of α provides a bridge to previous models, but the structural outcome also depends strongly on radial interactions and dynamic effects.

Magnetic relaxation—the finite time required for the magnetic dipole moments to align with the external field—plays a key role in our system. When the field direction is switched, the magnetization does not reorient instantaneously but instead lags behind due to this relaxation process. This delay introduces subtle but important long-time effects that influence the structure formation. In our experimental configuration, the primary field component *H_y_* always remains positive, while the perturbation component *H*_*x*_ alternates its sign between subcycles. Consequently, magnetic dipoles oscillate around the primary field direction due to relaxation. Each time the field direction changes, the dipoles realign accordingly, but with a small angular lag. This is particularly evident at high perturbation strengths. For the magnetic latex particles used here, the relaxation time is on the order of milliseconds,^63^ allowing direct observation of this rotational delay upon each field reversal. To illustrate this effect, we have included one supplementary video. Video S1 shows early stage evolution under *H*_x_ = *H*_*y*_, where small aggregates visibly swing around the primary field direction due to magnetic relaxation.

Though subtle, this swing around the primary field direction has long-term consequences on the aggregation process. Magnetic relaxation creates time-averaged anisotropic interactions that bias the aggregation along a preferential direction—similar to very recent finding by Biswal and coworkers under alternating rotational fields.^64^ This mechanism explains the formation of anisotropic clusters even under balanced fields (*H_x_* = *H_y_*), where isotropic structures might be expected. Notably, reversing the direction of the perturbation results in clusters aligned perpendicular to the main field. We elaborate on this behavior in Supporting Information S3, where we also demonstrate that fully isotropic (circular) structures emerge when both the primary and perturbation field components are alternated—i.e., when the field vector performs a full rotation.

Additionally, magnetic relaxation plays a key role in the rotational dynamics of the particles at the edges of the orthogonally percolating aggregates. The relaxation-induced rotational motion contributes to the continuous flattening of the banded structures over time. To illustrate this effect, we have included another supplementary video. Video S2 corresponds to late-stage evolution (after 50 min) at three different field frequencies (0.3, 1, and 3 Hz). In these cases, chains forming at the edges of the aggregates exhibit both fragmentation and rotation during realignment with alternating orthogonal fields. This effect is discussed in detail in Section 2.4.2

### 2.2 Phase Diagram

A deeper understanding of the field strength and frequency effect on the final structure can be obtained by scaling using the appropriate forces and times. While the field is on, the magnetostatic force, which drives particle coalescence in both directions, competes with Brownian motion, that tends to disperse particles more effectively in the perturbation axis. The magnetic coupling parameter λ allows us to quantify the balance between magnetostatic and thermal energy. It is defined as the ratio of the magnetic energy of two dipoles aligned tip-to-tip with the external field to the thermal energy.

4 $$\lambda =\frac{\mu_{0}\pi \sigma^{3}\beta^{2}H^{2}}{16k_{B}T}$$

Here, β = (μ_p_ - μ_0_)/(μ_p_ + 2μ_0_) is the magnetic contrast factor of the particles relative to the carrier medium. For small perturbations, thermal diffusion dominates during the primary field-off period, so the system behaves similarly to the UTF case, where the prevalent scale for structure coalescence and crystallization is the relation between the field frequency and the aforementioned thermal relaxation scale *t*_d_.^35^ Figure 3 shows phase diagrams after 3000 s for the three frequencies investigated (*f* = 0.3 Hz, 1 Hz, and 3 Hz, or 0.19*t*_d_^–1^, 0.65*t*_d_^–1^ and 2*t*_d_^–1^ respectively) as a function of λ along both the *X* and *Y* axes. The same four types of structures identified in Figure 2 are distinguished.

**Figure 3:**
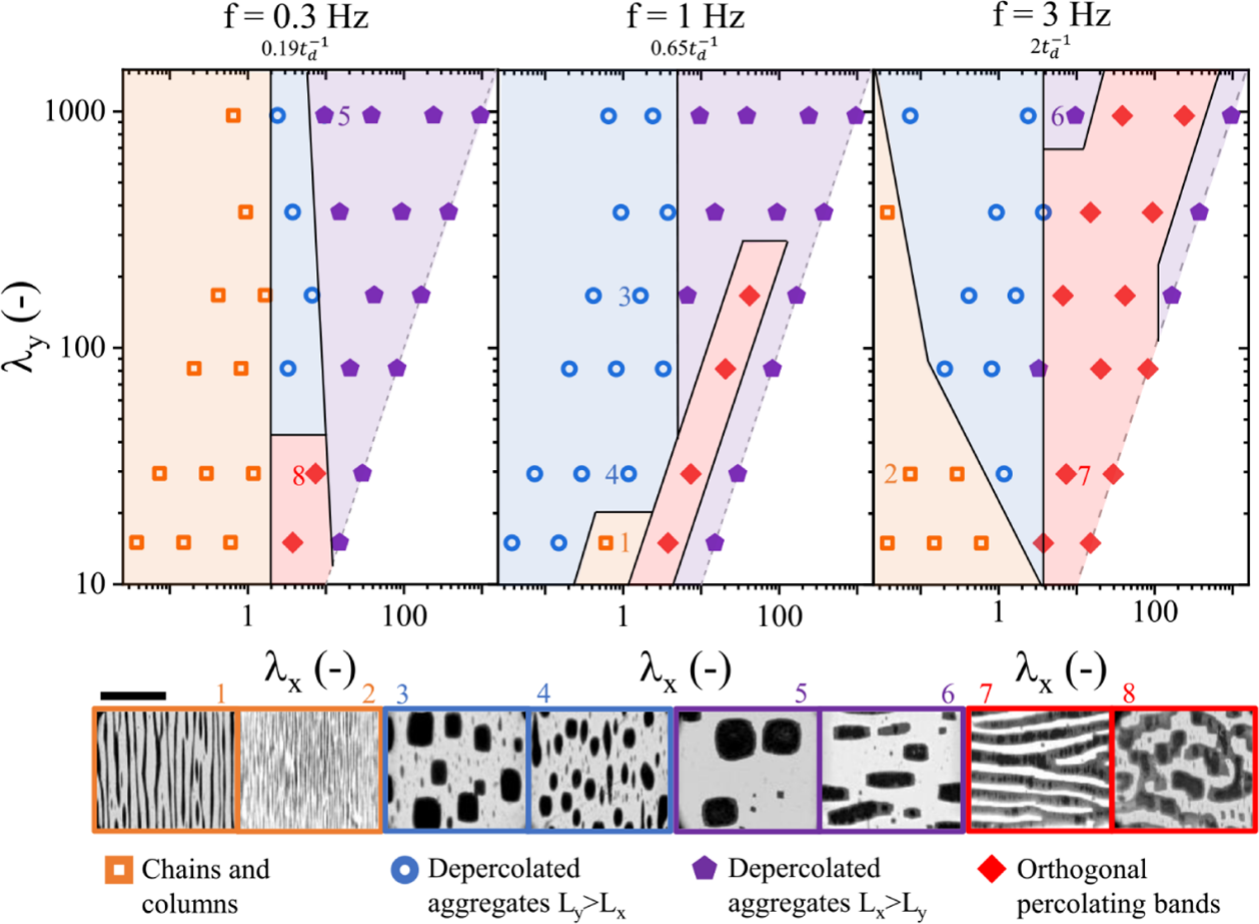
Microstructure phase diagram (obtained after 3000 s) as a function of the magnetic coupling parameter in primary λ*_y_* and perturbation λ*_x_* field directions at frequencies of *f* = 0.3 Hz, 1 Hz, and 3 Hz. Orange open squares correspond to percolating chains and columnar structures in the primary field direction. Blue open circles correspond to dense depercolated aggregates with their longest axis along the primary field direction. Purple pentagons correspond to dense depercolated aggregates with their longest axes oriented in the perturbation field direction. Red diamonds correspond to percolating bands in the perturbation field axis. Dashed lines represent the limit *H*_x_ /*H_y_* = 1. Black scale bar indicates 500 μm.

Across all frequencies, a structural transition occurs around a critical perturbation value of λ_*x*_ ∼ 1 , marking the shift from thermally driven to field-driven structures. For λ_*x*_≲ 1 the generated structures are extended along the primary field axis. We observed either percolating structures in the Y direction or dense, elongated aggregates with a longer semiaxis along the same direction, *L_y_* > *L_x_*. As discussed previously, due to the dominance of Brownian relaxation, structures are expected to be similar to the UTF case. As will be shown later (see Section 2.4), the key difference will be that the superposition of a perturbation field drastically accelerates the self-assembly process. Interestingly, at the highest frequency explored (*f* = 3 Hz), even weak perturbation fields can induce structure depercolation. In this case, the primary field-off period is too short for thermal motion to drive particle reorganization, but the orthogonal perturbation induces a slight diffusion, pushing the suspension toward a compact configuration. At low frequencies (*f* = 0.3 Hz), the enhanced diffusion due to weak perturbations intensifies the correlation loss between each field pulse, restricting the system to local energy minima (i.e., percolating chains or columns).

For λ_*x*_ > 1 the generated structures are predominantly oriented in the direction of the perturbation axis. In this regime, Brownian motion has minimal impact on the aggregation kinetics. At the lowest frequencies investigated (*f* = 0.3 Hz), most aggregates do not percolate and the longer structuring time allows for the formation of more compact structures through the stacking of particle layers in the direction of gravity (see Section 2.3 below). However, as the frequency increases (*f* = 3 Hz), percolating structures become more prevalent, as aggregates preferentially interact laterally rather than stacking vertically, resulting in the formation of percolating bands.

### 2.3 Crystallization

As discussed in the introduction, monodisperse MCs are expected to form crystalline structures under the application of toggled fields. Figure 4 depicts close-up images and their Fast Fourier Transforms (FFTs) of the different structures identified in the phase diagram shown in Figure 3. For completeness, a micrograph of a MC structured under a typical uniaxial steady field is also shown.

**Figure 4:**
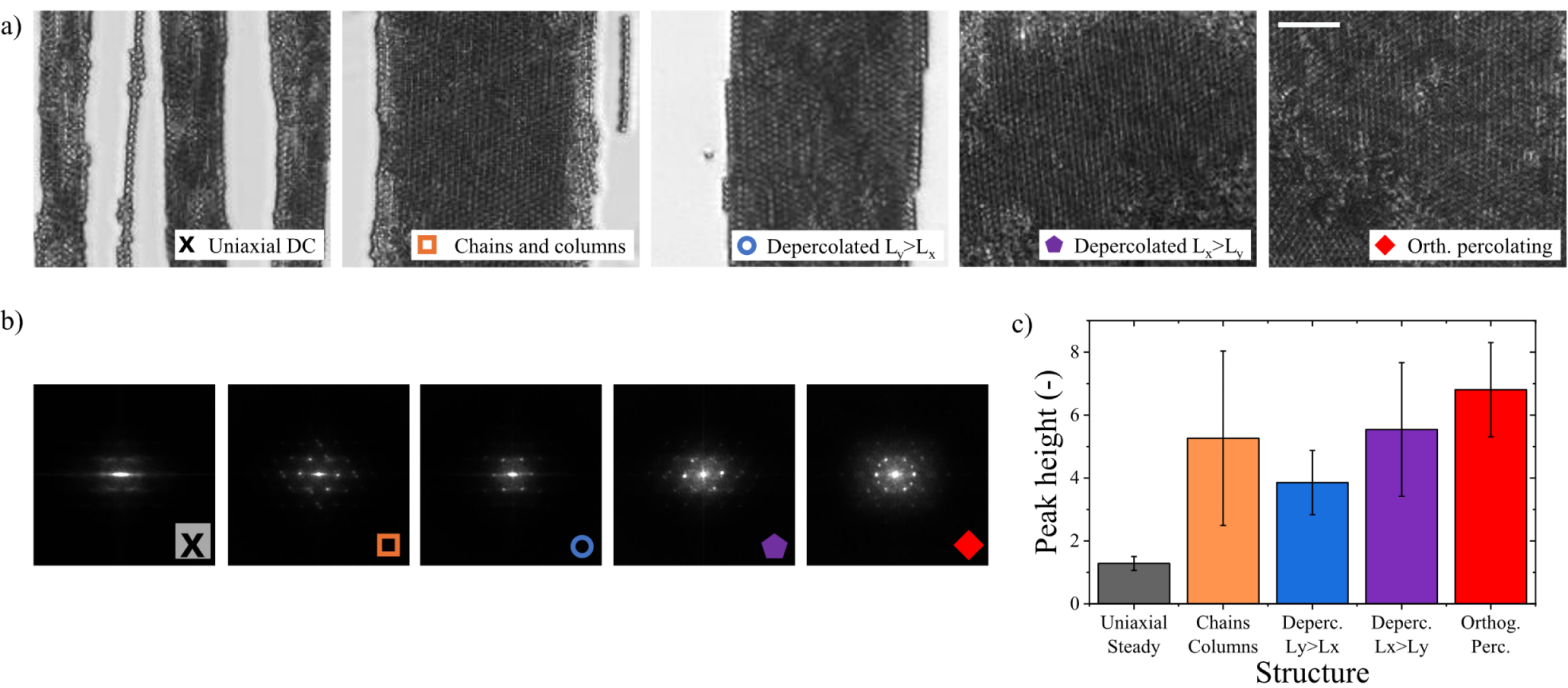
(a) High-magnification microscopy images corresponding to the four types of structures observed in Figure 3. While uniaxial steady fields result in kinetically arrested, defect-laden open structures, biaxial toggled fields (BTFs) promote a phase transition to an ordered body-centered tetragonal (BCT) arrangement. The white scale bar represents 10 μm. (b) Fast Fourier Transform of the microscopy images reveal the ordered particle arrangement. (c) Average intensity of the six most prominent FFT peaks, demonstrating a significantly lower degree of crystallinity in the steady field case.

Under a uniaxial steady field, MCs do not form crystal like structures (see Figure 4a). This occurs despite thermodynamic equilibrium theory predicting that a dipolar suspension under a sufficiently strong continuous field should condense into crystalline domains.^65^ The discrepancy arises because, under strong fields, particles quickly assemble but remain trapped in a kinetically arrested metastable state, residing in a local energy minimum far from the optimal configuration.^50,66^ The limited mobility of the particles due to the strong, steady field results in the formation of loose, open structures with numerous defects.

In contrast, BTFs drive MCs to form crystal-like structures (see Figure 4a), with large crystalline domains exhibiting few defects. The enhanced particle mobility and lateral aggregation induced by the BTF effectively overcome kinetic barriers, allowing the system to achieve a highly crystalline structure much closer to the absolute energy minimum. Experiments under UTFs^33,50,67^ and rotatory fields^40–43^ have reported crystalline structures corresponding to body-centered tetragonal (BCT) lattices viewed from the (110) plane, consistent with thermodynamic theory predictions for the employed field strengths and concentrations.^65,68^ Under BTFs BCT lattices aligned with the main field direction are also observed in the low perturbation limit; however, strong perturbations may favor other hexagonal lattices, as discussed further below in this section.

The FFT of the micrographs shown in Figure 4a was also conducted to quantify the crystallinity of the observed structures. The results are shown in Figure 4b. The aggregates formed under a uniaxial steady field exhibit low crystallinity, as evidenced by weak, diffuse intensity peaks, while the large peak width indicates a small crystalline domain size. In contrast, the formation of large crystalline domains under BTFs is demonstrated by the presence of high-intensity, sharp peaks with 6-fold symmetry. Notably, additional rotated intensity peaks emerge, signaling the presence of large crystalline domains with different orientations. This polycrystallinity is also visible in the micrographs shown in Figure 4a. Finally, Figure 4c displays the average intensity of the six main peaks, highlighting the significantly lower degree of crystallinity in the steady field case.

To investigate the effect of perturbation strength on the resulting crystalline structure, additional videomicroscopy experiments were performed. The final crystal structure depends on both the absolute field strength and the relative ratio between perturbation and primary fields. In our experiments, we explored the ranges *H_y_* = [1.5–12] kA·m^–1^ (corresponding to λ_*y*_ = [15–1000]) and *H_x_*/*H_y_* = [0.05–1] (corresponding to λ_*x*_/λ_*y*_ = [0.0025–1]). These conditions ensure that magnetic interactions dominate over thermal motion along the primary field direction, but this is not necessarily the case for the perturbation direction.

In Figure 5 we show micrographs of the internal structure for a perturbation strength sweep, where we distinguish two distinct regimes. For small perturbations­(λ_*x*_ ≲ 1), the resulting crystal structure is a BCT lattice aligned along the primary field direction (*H*_*y*_). In this regime, thermal motion can be comparable to or exceed the perturbation field. Consequently, all crystalline domains align with *H*_*y*_, consistent with UTF behavior. The perturbation field and thermal energy facilitate structural relaxation, but not reorientation. This is consistent with prior theoretical predictions,^65,67^ which indicate that for uniaxial fields, the lowest energy configuration is a BCT lattice with its short axis aligned with the field.

**Figure 5:**
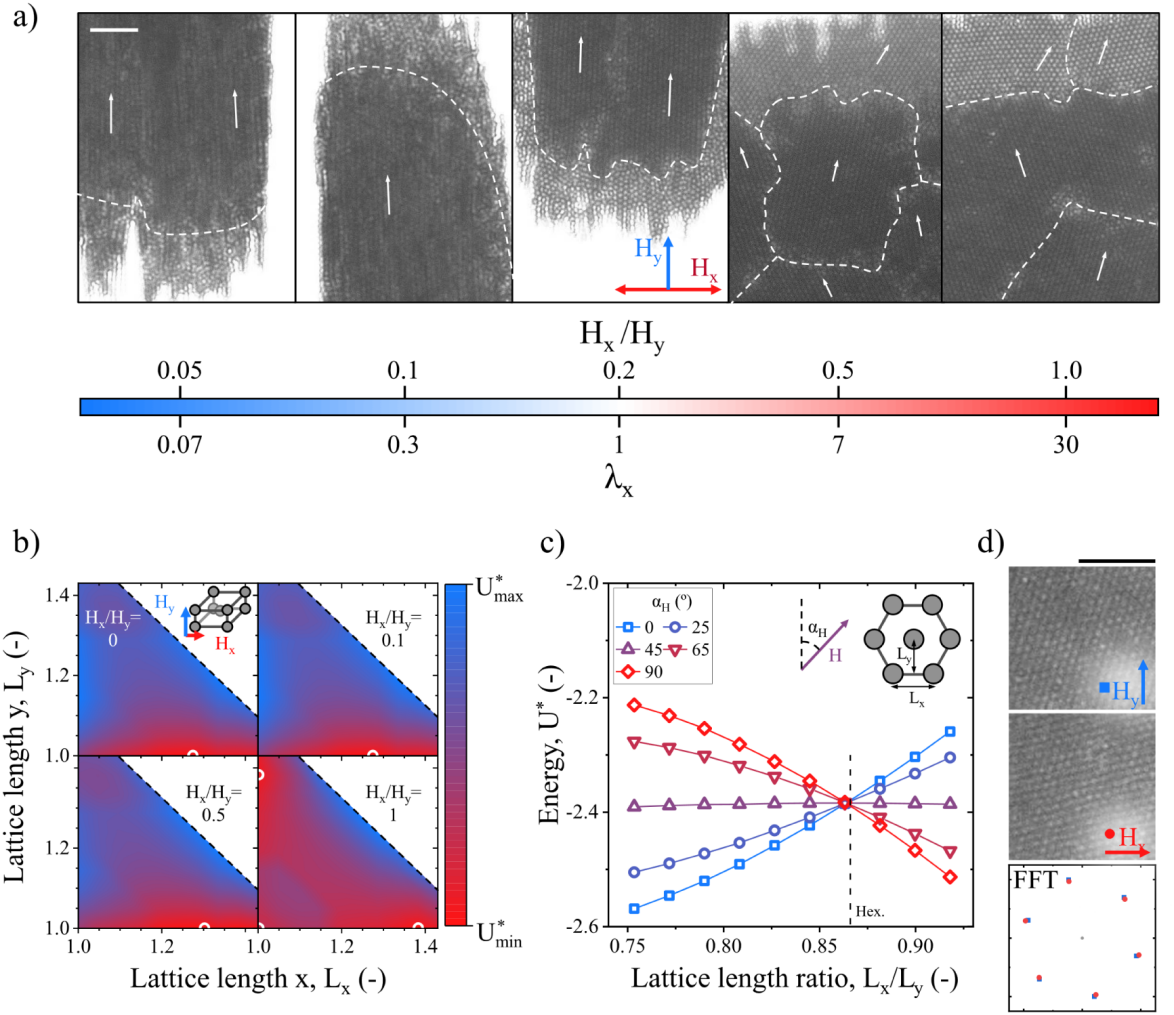
Analysis of the crystalline microstructure. (a) Internal microstructure as a function of the perturbation strength. The color bar indicates both the relative and absolute strength of the perturbation field, with respect to a fixed primary field of *H*_y_ = 2.1 kA·m^–1^ (λ_*y*_ = 30) toggled at a frequency of *f* = 1 Hz. Crystalline domain boundaries and their respective orientation are marked. All micrographs were captured at time points when the primary field was active. (b) Energy landscape of a BCT lattice under two orthogonal dipolar fields of strengths *H*_*y*_ and *H_x_*. For weak perturbations, the energy minimum—pointed with white circles—remains largely unchanged, favoring configurations where the lattice is contracted along the primary field direction. For strong perturbations, two absolute minima appear, corresponding to lattice contraction along one of the axes, and a relative minima signaling contraction along both *X* and *Y* axes. (c) Energy landscape for a 2D hexagonal lattice under a magnetic field aligned at different angles α_H_ and different lattice length ratios *L*_*y*_/*L*_*x*_. The most favorable states are lattices compressed along the field axis in all cases. Dashed line marks the perfect hexagonal lattice. (d) Micrographs from an experiment with *H*_*y*_ = *H*_*x*_ = 2.1 kA·m^–1^ and *f* = 1 Hz during application of *H*_*y*_ and *H_x_*, respectively. In the bottom subfigure, the position of the main peaks of the FFT of both structures is shown, revealing the stretching of the lattice in orthogonal directions. Scale bars represent 10 μm in all cases.

We also carried out an energy analysis similar to Sherman et al. (2018)^67^ for the case of biaxial toggled fields by superimposing two orthogonal dipolar potentials and evaluating energy minima across a range of unit cell geometries. Details on energy calculations can be found in the Materials and Methods section. The resulting energy landscape, shown in Figure 5b for different perturbation strengths and dimensions of the unit cell, reveals that weak perturbations have a negligible effect; the BCT structure still exhibits a clear energy minimum when its short axis aligns with the primary field direction. In contrast, in the strong perturbation regime (λ_*x*_ > 1) the energy analysis reveals two distinct minima: one where the lattice is maximally contracted along *H_x_*, and another along *H_y_*, with a relative minimum at intermediate contraction along both axes. These predictions match experimental observations. Under strong perturbations (i.e., λ*_y_* > 1 and λ_*x*_ > 1), the internal structure no longer exhibits a uniformly aligned crystalline phase. Instead, we observe polycrystalline hexagonal domains with varying orientations (see Figure 5a), as reported in rotating magnetic fields.^69^ These domains dynamically realign, especially at edges (see Video S2), reflecting strong dipolar interactions along both field directions. Micrographs clearly show the boundaries between differently oriented domains.

Moreover, each crystalline domain exhibits a slight anisotropic deformation depending on the instantaneous field direction. The lattice contracts along the active field direction at each toggle. To quantify this effect, we extended our energy calculations to stretched 2D hexagonal lattices arbitrarily oriented with respect to the field an angle α_H_. As shown in Figure 5c, the energy is minimized when the lattice is contracted along the field axis, in line with experimental results. These calculations were performed under the assumption of hard spheres with a 2D packing fraction ϕ_2D_ = 0.85 similarly to Hynninen and Dijkstra (2005).^65^

We experimentally validated this deformation by analyzing the FFTs of micrographs captured under fields oriented along *H_y_* and *H_x_*. As illustrated in Figure 5d, the FFTs reveal consistent lattice contraction along the field direction. Averaging over multiple regions and cycles, we measured an anisotropy in lattice spacing, *L*_*y*_/*L*_*x*_ = 5.4 ± 0.8%, for both *H_x_*/*H_y_* = 0.5 and *H*_x_/*H*_y_ = 1, with no significant difference between these two perturbation strengths.

It is important to emphasize that these energy calculations provide qualitative insights under the assumption of quasi-equilibrium conditions. In the strong-field regime, field toggling induces continuous particle rearrangements, driving the system out of equilibrium. Nonetheless, the trends captured by the energy landscape are consistent with experimental observations and support the interpretation of structure transitions as a function of field strength and symmetry.

Finally, we note that the orientation of the crystalline domains can be tuned by adjusting the relative angle between the applied fields. Applying the perturbation field along specific crystallographic directions (e.g., 60° in the case of hexagonal lattices) promotes domain alignment. This concept is explored in detail in Supporting Information S4.

### 2.4 Coarsening Kinetics

In this section, we investigate how the different structures reported grow under the influence of the perturbation field. Two different methods are used to analyze the micrographs, as described in Materials and Methods section. The first method utilizes the spectral power density to determine the characteristic length in the direction of the perturbation field. This is achieved by taking the inverse of the wavenumber corresponding to the peak of the power spectrum *L*_char_ = 2π/*q*_max_. This approach has been used in previous studies involving UTFs^32,33,35,70^ and in light scattering investigations of particle chaining.^71–74^ The second method calculates the mean cluster size through aggregate segmentation and tracks its evolution over time, a technique commonly used in videomicroscopy studies under steady magnetic fields.^16,75–77^

#### 2.4.1 Characteristic Length

Analyzing the spectral power density helps determine the characteristic length of the aggregates along the X direction. For small *H_x_*, the perturbation field’s influence on the final structure remains minimal compared to UTFs^35^ (see Section 2.1). However, it significantly impacts growth dynamics. The addition of the perturbation field during the primary field-off period clearly accelerates the self-assembly process, as illustrated in Figure 6, which depicts the characteristic length *L*_char_ derived from the inverse of the spectral maximum wavenumber. Data is shown for various *H_y_* values, with and without a perturbation field of small amplitude (*H_x_*/*H_y_* = 0.1). The phase separation rate increases with the strength of the primary field *H_y_*. For small *H_y_*, a two-step coarsening is observed: an initial subdiffusive regime *L*_char_ ∼ *t* ^0.25^, followed by a ballistic regime *L*_char_ ∼ *t* after a critical time *t*_c_. As expected, this behavior is similar to the one observed in UTFs because λ_*x*_ ∼ 0.1 < 1. In contrast, for intermediate and high *H_y_*, the growth follows a single step coarsening process described by *L*_char_ ∼ *t* ^0.5^. This 0.5 exponent scaling has been reported in other systems^78–80^ where coarsening results from deep quenches^81^ due to pronounced asymmetries in relaxation time scales between the particle-rich and poor phases. Therefore, in BTFs, the transition from two-step to single-step coarsening takes place at lower *H_y_* than in UTFs, primarily due to stronger lateral interactions induced by the secondary field. In practical terms, this accelerated assembly enables more efficient processing and potential cost reductions, which enhances the method’s applicability for scalable material fabrication.

**Figure 6:**
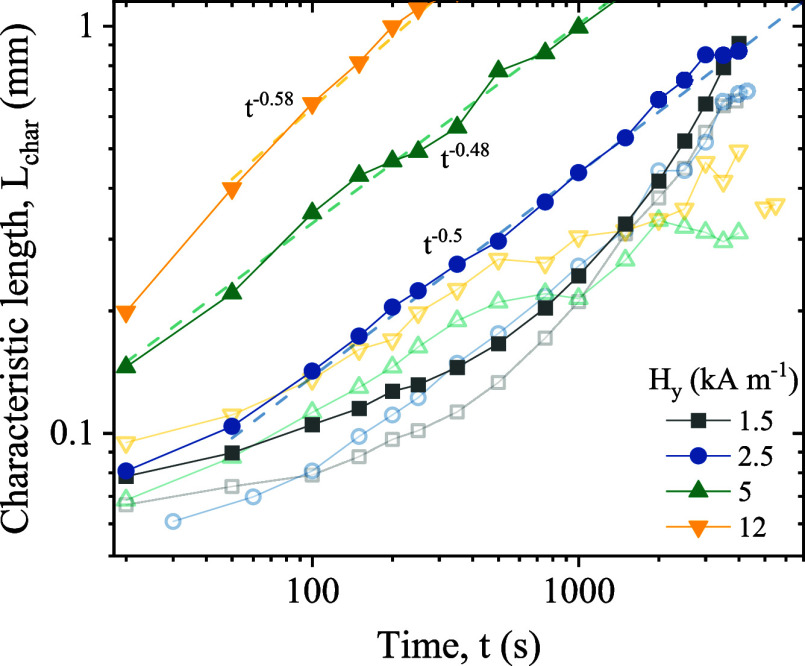
Characteristic length *L*_char_ as a function of time t for BTFs with a perturbation strength of *H_x_*/*H_y_* = 0.1. Open symbols correspond to experiments under UTFs.

The observation that most structures formed with BTFs (except those with dominant Brownian motion) exhibit growth following a power law *L*_char_ ∼ *t* ^0.5^ suggests the possibility of defining a scaling time to consolidate the results into a master curve. Indeed, the initial growth dynamics are governed by the aggregation time *t*_a_ provided that magnetostatic interactions prevail over Brownian motion.^16^ Therefore, since *L*_char_ quantifies the aggregate size along the perturbation field direction, the relevant aggregation time can be expressed as follows:

5 $$t_{a,x}=\frac{2\eta }{5\mu_{0}\beta^{2}H_{x}^{2}}{\left(\frac{\pi }{6\phi_{2D}}\right)}^{5/2}$$

This aggregation time scale *t*_a,*x*_ is defined as the duration needed for two particles within a suspension of surface fraction ϕ_2D_ and viscosity η to merge due to dipolar magnetostatic interactions. This definition facilitates the introduction of a dimensionless, reduced time, t* ≡ *t*/*t*_*a*,x_ that captures, for instance, the temperature dependence of the viscosity. Figure 7a displays all characteristic lengths from the experiments, with each color and symbol indicating different field frequencies and the color gradient representing the strength of the perturbation field. The main trend observed is an accelerated aggregation rate as both primary and perturbation field strengths increase, consistent with previous results in UTFs.^82^ Figure 7b presents the data scaled by the aggregation time, demonstrating a reasonable data collapse, except for extreme cases of perturbation field strength. Two distinct growth regimes emerge. On the one hand, a short-time regime characterized by subdiffusive growth. This region aligns with small field strengths λ_*x*_ < 0.15 and λ_*y*_ < 25), where thermal diffusion predominates over the perturbation field. On the other hand, a long-time regime, identified at higher field strengths, exhibiting the aforementioned 0.5 scaling exponent associated with strong attractive interactions.^81^ Thus, a master curve is constructed, that is valid across a broad range of fields intensities, perturbations, and frequencies. However, as shown in Figure 7, at very high fields deviations occur; for very strong fields the initial aggregates repeatedly fragment and reorient due to prolonged field-on periods compared to the aggregation time, thereby constraining early stage growth. The influence of particle surface fraction on the characteristic length scale dynamics is found to be negligible as demonstrated in Supporting Information S5.

**Figure 7:**
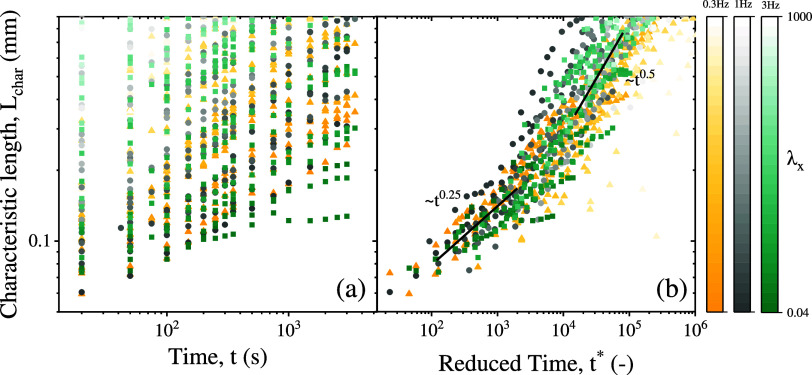
(a) Characteristic length *L*_char_ of the structures for all the experiments performed. Yellow triangles correspond to *f* = 0.3 Hz, black squares to *f* = 1 Hz and green circles to *f* = 3 Hz. The color gradient indicates the perturbation strength. (b) Master curve showing the characteristic length of the structures *L*_char_ as a function of the reduced time t* = *t*/*t*_a,*x*_. For weak perturbations a diffusion-limited regime is identified (*L*_char_ ∼ *t* ^0.25^). A deep quench regime is found (*L*_char_ ∼ *t* ^0.5^) when the magnetic perturbation dominates over Brownian motion.

#### 2.4.2 Mean Cluster Size

The use of the power spectrum for structural characterization is inherently limited by the number of aggregates visible within the micrographs. Specifically, the maximum spatial scale that can be calculated using this method is considerably smaller than the full field of view, as periodicity in the analyzed direction is required to detect the peak at *q*_max_. Additionally, this method primarily captures length scales along the direction of the perturbation field, whereas the observed structures exhibit complex morphological changes along the primary field axis as well. To address this limitation and gain further insights into the evolution kinetics, the weighted average cluster size was also calculated using the formula $S\left(t\right)=\frac{\underset{s}{\sum }s^{2}n_{s}\left(t\right)}{\underset{s}{\sum }sn_{s}\left(t\right)}$ , where *n*_*s*_ represents the number of clusters with surface area *s*. Additional details on image analysis and cluster size calculation are provided in Materials and Methods section.

Dynamic scaling theory (DST) provides a framework for understanding how the average aggregate size evolves over time, expressed by the power law *S*(*t*) ∼ *t^z^*, where *z* is the kinetic exponent.^83^ DST has been applied effectively to describe the aggregation process in MCs at low concentrations under uniaxial steady magnetic fields, where isolated single-particle width chains form primarily by tip-to-tip aggregation.^84^ In the thermally driven diffusion regime, the kinetic exponent is around *z* ≈ 0.5 while in non-Brownian systems, strong dependencies with λ and ϕ seem to exist.^75^ Deviations from the predicted exponent of 0.5 have also been explained in terms of friction with the confining walls.^16^ The interpretation of DST for aggregates formed under BTFs is far more complex; aggregates have diverse morphologies, multiple layers in the gravity direction and interconnections.

Figure 8 illustrates the structural evolution for selected cases within the four regimes identified in Figure 3. Accompanying the micrographs are plots of mean cluster size *S* as a function of time *t*. Supporting Information S6 shows a wide range of *S*(*t*) curves across different field strengths and parameter combinations to support the representativeness of the selected curves.

**Figure 8:**
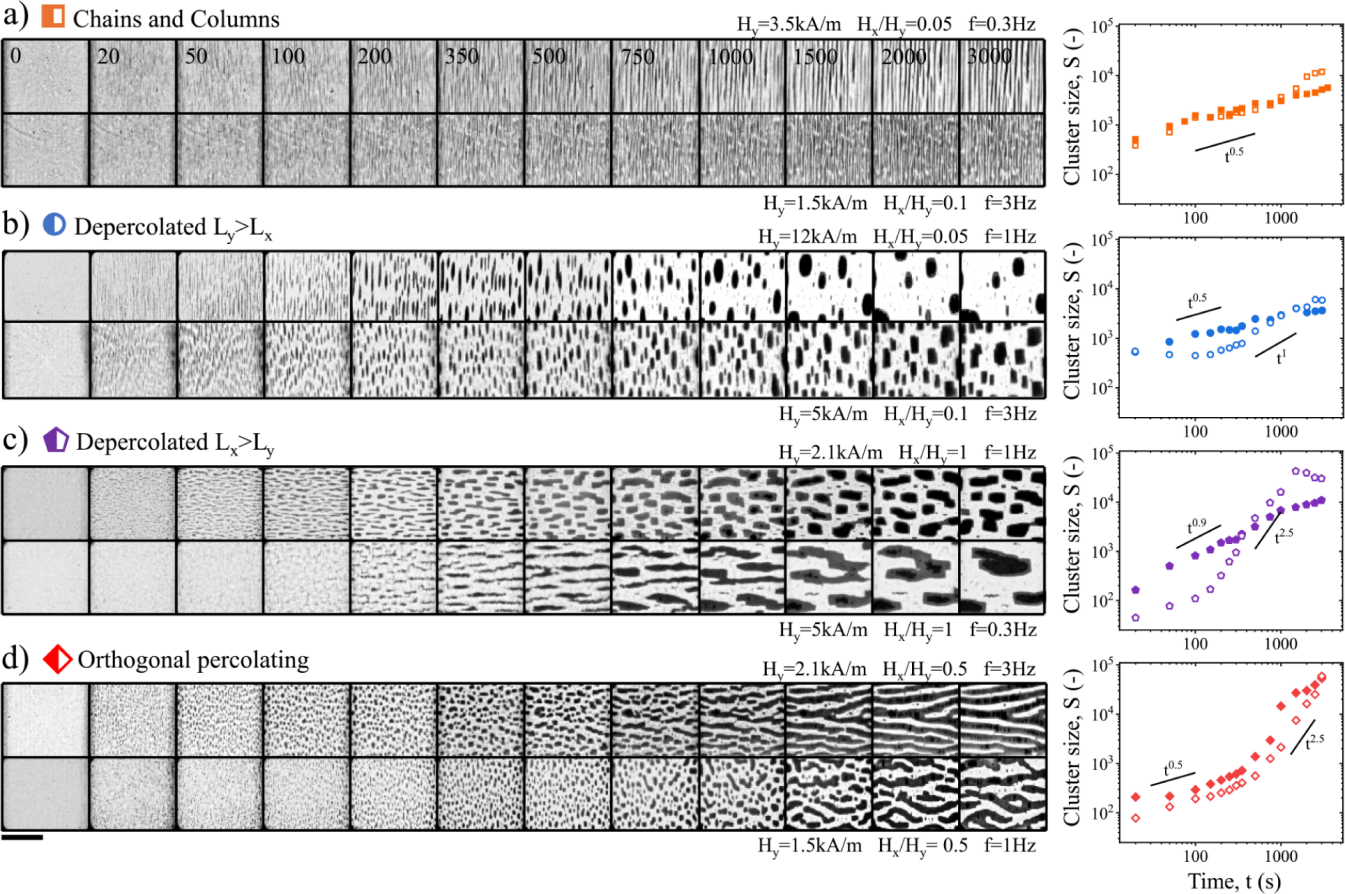
Time-lapse snapshots of the structures corresponding to the four regimes identified in Figure 3. The graphs depict the time evolution of the weighted average cluster size *S*(*t*) with filled symbols representing data from the first row and empty symbols representing data from the second row for each structure. Characteristic scaling trends are included for visual reference. Black scalebar indicates 500 μm.

In the vertical chains and columns regime (Figure 8a), a consistent growth dynamic with an exponent of approximately *z* ∼ 0.5 is observed. At the lowest frequency (*f* = 0.3 Hz), the resulting columns are thick with minimal lateral interconnections. However, at higher frequencies (*f* = 3 Hz), the dynamics resemble those observed in uniaxial steady fields.^35^

In the vertical depercolated regime, we observe a rapid growth dynamics (see Figure 8b), with cluster sizes increasing at a kinetic exponent in the range 0.5 < *z* < 1. At the early stages, stretched aggregates are quickly formed along the primary field direction, which later contract along this axis and expand horizontally due to lateral merging. Notably, much sharper aggregate boundaries emerge at the higher frequency (*f* = 3 Hz).

In the horizontal depercolated regime growth dynamics are highly dependent on the magnetic field strength along both axes (see Figure 8c). At low field strengths, structures remain cohesive during each structuring semiperiod, showing a monotonous growth dynamic. In this case, the perturbation field promotes the formation of elongated aggregates in the horizontal direction at early times, that then coalesce into more compact structures. Conversely, at high field strengths, particles are able to rearrange during each semiperiod, forming small aggregates with chains of a few particles length that orient in the instantaneous field direction. These small aggregates are free to rotate; however, as assembly progresses, the formation of larger aggregates restricts the movement of the smaller chains, preventing internal particle reorientation in each semiperiod and thus accelerating overall growth. Consequently, for intense magnetic fields, we observe a transition from slowly growing small initial aggregates to rapidly growing larger aggregates. In this scenario, perturbation is strong enough to form percolating bands in the early stages, making the dynamic comparable to orthogonal bands, as discussed below. However, in the later stages of assembly, strong attractive interactions favor the stacking in the gravity direction, thus tearing apart the percolating aggregates, reflected in the slight decrease in aggregate area at late stages in Figure 8c.

Finally, the orthogonal percolating bands regime exhibits one of the most intriguing dynamics (see Figure 8d). Initial growth follows a pattern similar to the other three regimes, but after a certain critical time, the growth rate increases dramatically, with a kinetic exponent of *z* ∼ 2.5. This rapid growth was not captured by the power spectrum analysis, as the structures become so large that they span the sample volume, making it impossible to measure a characteristic length from the image. Over longer time scales, a zigzagging pattern emerges due to vertical interaggregate interactions under the primary field. This pattern gradually flattens as the aggregates minimize surface magnetic energy,^62^ driven by the rotational dynamics of the particles at the edges of the aggregates [similar to^58^ and^62^]. These band structures, along with their dynamics, are also very similar to those observed by Kim et al. (2020)^32^ in horizontal pulsed fields under specific field strengths and duty ratios. Under appropriate conditions, the perturbation field can act as an analog to controlling the duty ratio in the case of UTFs, driving the lateral aggregation of particles.

This rotational dynamic has been characterized through high-resolution video microscopy experiments. Observations at the edges of the aggregates reveal the presence of short particle chains that reorient periodically with the direction of the applied field. Upon each orthogonal switching of the field, these chains partially rotate due to magnetic relaxation effects (as discussed in Section 2.1) and transiently disassemble before realigning in the new field direction, as shown in Supplementary Video SV1. This continuous reorientation generates net particle migration along the aggregate surface and underlies the rotational dynamics.

The direction of rotation depends on the inclination of the band. As illustrated in Figure 9a, horizontal bands present no lateral asymmetry in accessible space for particle motion during reorientation, leading to negligible net displacement. In contrast, tilted bands (Figure 9b) exhibit an asymmetric distribution of accessible space due to their orientation relative to the primary field direction. This asymmetry generates a preferred direction of particle movement: bands with a positive slope rotate counterclockwise, while those with a negative slope rotate clockwise.

**Figure 9:**
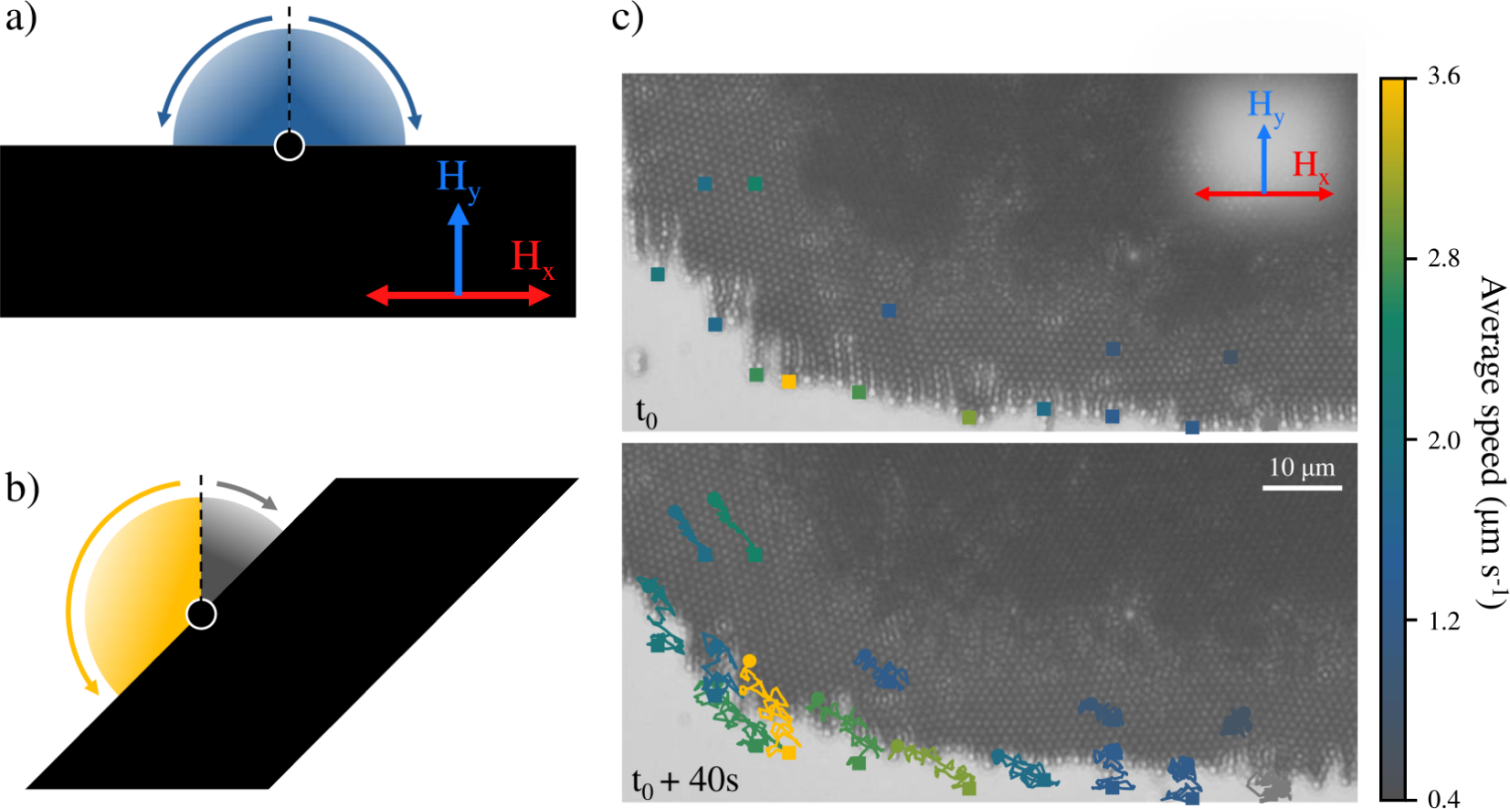
Rotational dynamics at the band edge. (a, b) Schematic of the rotation dynamics mechanism. In a flat band (along the perturbation direction), small chains at the edge can rotate and reorient equally to both sides, resulting in no net motion. In contrast, in a band with a finite slope, more available space in one direction guides the particle motion preferentially toward that side. (c) Experimental particle dynamics at the orthogonal band edge. The top image shows the initial positions of the tracked particles, marked with square symbols at *t*0 = 3000 s of structuration. The bottom image displays the particle trajectories after 40 s, with final positions indicated by circles. The color of each trajectory corresponds to its average speed. Experimental conditions: *H_y_* = 2.1 kA·m^–1^, *H*_*x*_/*H*_*y*_ = 0.5, *f* = 0.3 Hz. Note that the field frequency was kept low to prevent particle loss during tracking.

This mechanism is confirmed by particle tracking experiments at the edges of differently inclined regions within the same aggregate (Figure 9c). In the flat horizontal section (bottom right), tracked particles display only small fluctuations around their initial positions, with net displacements yielding average speeds between 0.4 and 1 μm/s—effectively negligible and likely attributable to slow drift of the aggregate as a whole. In contrast, in the negatively tilted region (bottom left), particles exhibit clear clockwise migration along the aggregate edge, with average net speeds ranging from 2 to 4 μm/s. This quantifies the rotational motion and demonstrates its dependence on local inclination.

Prolonged self-assembly experiments show that these orthogonal bands, while not undergoing further coarsening in the main field direction, display a long-term flattening along the perturbation direction as a consequence of the rotational dynamics (see Supporting Information S7 for micrographs of evolution up to 8500 s). This phenomenon also explains the accelerated aggregate flattening observed at higher frequencies (see Figure 2). At the highest frequencies explored, the rotation and realignment occurring at each field switching—which drives particle motion along aggregate edges—occurs more frequently. Consequently, particles reach flat regions and achieve steady configurations more rapidly. The band periodicity of quasi-steady configurations is strongly dependent on the main field strength, and exhibits a linear relationship with *H_y_* (see Supporting Information S8).

## 3 Conclusions

To summarize, we introduce a novel approach to accelerate phase separation dynamics and promote the formation of tunable structures in magnetic colloids by superimposing orthogonal toggled fields within the aggregation plane. Instead of relying on Brownian motion, a perturbation field is employed to enhance the lateral aggregation of the structures, reducing the repulsive region and hindering the pair rotation of the dipolar interaction force. At low field strengths, rapid self-assembly occurs, while at high field strengths, unique 2D structures with controllable aspect ratios can form, ultimately leading to the creation of percolating bands.

Based on the structures formed over long periods, we identify four distinct regimes, which are strongly influenced by field frequency and strength along both axes. These regimes can be mapped onto a phase diagram defined by the magnetic coupling parameter: vertical chains and columns, vertical depercolated aggregates, horizontal depercolated aggregates, and orthogonal bands. The internal microstructure of the aggregates exhibits a highly compact crystalline structure, which contrasts sharply with the structures formed under traditional steady fields. At the same time, the nonequilibrium nature of the system introduces rich rotational dynamics at the edges of the aggregates that drive structure evolution.

The growth dynamics are found to vary depending on the specific regime. A master curve is proposed that allows for the determination of the desired characteristic length of the aggregate at appropriate scaling times. These diverse structures and dynamics open up new possibilities for applications, as the different internal microstructures influence responses to external stimuli. For instance, by precisely controlling percolation, the rheological response can be significantly modified.^28^ In photonic materials, where high-quality crystals of controllable size are often desirable, biaxial toggled fields may offer a potentially valuable approach,^85^ alongside other emerging strategies in the field of smart materials.^86^ Furthermore, given the dynamic nature of the structure, which undergoes sustained growth over extended periods, any internal microstructure of desired size can be achieved by preserving the aggregates at any stage of the assembly process through rapid freezing of the carrier medium. This technique could be used to produce composites with optimal physicochemical properties,^21,87,88^ and structured colloidal hydrogels that could serve as scaffolds for guided cell growth.^89–91^

## 4 Materials and Methods

### 4.1 Sample Preparation

Magnetic colloids (MCs) used in this work were prepared by dispersion of magnetic latex particles with a σ =1 μm diameter (Dynabeads MyOne Carboxylic Acid, ThermoFisher) in water (viscosity, η =1 mPa·s). The particles remained stable without any degradation or oxidation for several months. For all the experiments reported in this work, the particles magnetization operated in the linear magnetostatic regime. Magnetization curve of the particle powder is available in Supporting Information S9. Table 1 contains relevant physical properties of the particles and MCs. MCs were placed in a homemade cylindrical holder with a glass bottom surface. Prior to the experiment, the MCs were left to sediment for sufficient time for the particles to form a monolayer on top of the glass bottom surface. Unless otherwise specified, the surface occupation was ϕ_2D_ = 0.45. Additionally, sodium dodecyl sulfate was added in a very dilute concentration (1 mg/mL) to reduce friction with the bottom wall of the sample holder.^16^ The samples remained fully hydrated throughout the experiments.

**Table 1:** Physical Properties of the Magnetic Latex Particles and MCs^a^

**Suspension properties**	
Mean particle size,σ (μm)	1.05
Polydispersity index, PDI (−)	0.004
Particle density, ρ_P_ (g·cm^–3^)	1.8
Volume fraction,*ϕ* (−)	3.9 × 10^–6^
Surface fraction, ϕ_2D_ (−)	0.45
Diffusion coefficient, *D* (m^2^·s^–1^)	4.4 × 10^–13^
**Magnetic properties**	
Initial magnetic contrast factor, β	0.31
Saturation magnetization, *M*_sat_	42.5 kA·m^–1^
Saturation magnetic field, *H*_sat_	100 kA·m^–1^
**Characteristic time scales**	
Sedimentation time, *t*_g_ (min)	21 min
Diffusion time, *t*_d_ (s)	0.65
Aggregation time, *t*_a_ (s)	0.003 (λ =13)
	4.6 × 10^–5^ (λ = 820)

a The PDI is calculated from 241 particles using PDI = (SD/AD)^2^ where SD is the standard deviation and AD is the average diameter

### 4.2 Field Generation

The magnetic field was generated through a custom built triaxial magnetic field generator constituted by five coils with mumetal cores in orthogonal configuration.^49^ In the experiments conducted in this study, only the four coils located in the XY plane were activated, and operated at low frequency. To generate squared toggled fields we used a signal correction applying a voltage overshoot at both the rising and falling edges of the pulse to get a pulse-like signal in the current and the magnetic field (see Tajuelo et al. 2023 ^49^ for further details on correction parameters). Alternative pulses were generated in perpendicular directions to obtain the desired BTFs. Figure 1a shows a schematic representation of the magnetic field generator, Figure 1b depicts the field configuration used and Figure 1c represents the voltage overshoot correction scheme. The *Y* field component (*H_y_*) is the primary field component, while the orthogonal toggled field (along the *X* axis, *H_x_*) is defined as the perturbation or secondary field. Note also that opposite successive pulses were applied in the *X* axis to avoid out-of-axis time-averaged fields and to avoid any symmetry breaking in the perturbation axis. The field frequencies used in this study are sufficiently low that magnetothermal effects can be considered insignificant.

### 4.3 Imaging

Large field-of-view images were obtained using a Leica Z6 APO stereomicroscope in upright configuration and a high-speed camera Photron MiniAX (18×), to track the suspension dynamics. For better illumination, an Effilux plain lamp was placed underneath the sample. Detailed information on the internal microstructure of the aggregates was obtained coupling the magnetic field generator either to a Zeiss Axio Observer microscope equipped with an Axiocam 305 camera and a 20× objective, or a Leica DMI3000 B with a 100× objective.

### 4.4 Image Analysis

Analysis of the recorded micrographs was carried out using ImageJ and a Python script. The power spectrum of the images was calculated along the *X* axis direction to study the growth of the aggregates in the perpendicular direction to the primary axis. The characteristic length of the structures *L*_char_ was calculated from the maximum wave vector *q*_max_ using *L*_char_ = 2π/*q*_max_. Given that the power spectrum is a noisy function, to reduce noise in the characteristic length determination, the first moments of the spectrum function were used, with $q_{\max}=\frac{\int qI\left(q\right)dq}{\int I\left(q\right)dq}$ .^72^ The total cluster area was also computed by a segmentation algorithm. The weighted average cluster size was calculated using $S\left(t\right)=\frac{\underset{s}{\sum }s^{2}n_{s}\left(t\right)}{\underset{s}{\sum }sn_{s}\left(t\right)}$ , where *n*_*s*_ is the number of clusters with surface area *s*. The surface area *s* is expressed in units of particle size, that is, as the ratio of the cluster area to a single particle area, *s* =*A*_clust_/*A*_part_. By evaluating *L*_char_(*t*) and *S*(*t*) evolution, we were capable to track the suspension dynamics and elucidate how the aggregation proceeded. Lastly, the internal crystalline structure of the aggregates was evaluated from the high-resolution microscope images by performing its 2D Fourier transform and identifying the crystalline periodicity peaks and their heights.

### 4.5 Energy Calculations

Thermodynamically favorable crystalline structures were determined through energy optimization, considering hard sphere repulsive potential and magnetic interactions within the dipolar approximation:^65^

$$U_{ij}^{\mathrm{dip}}=\frac{\mu_{0}}{4\pi r_{ij}^{3}}\left[{\mathrm{m⃗}}_{i}\cdot {\mathrm{m⃗}}_{j}-3\left({\mathrm{m⃗}}_{i}\cdot {\mathrm{r̂}}_{ij}\right)\left({\mathrm{m⃗}}_{j}\cdot {\mathrm{r̂}}_{ij}\right)\right]$$

where *m⃗*_*i*_ is the magnetization of a particle *i*. In our specific case, we neglect mutual magnetization, considering only magnetization by the external magnetic field. This assumes that each particle acquires an identical dipole moment equivalent to that of a single isolated particle (*m⃗_i_* = *m⃗*_*j*_ = πσ^3^β^2^ *H⃗*_ext_^2^/2). This approximation is strictly valid only in the low *β* regime, while incorporating mutual magnetization provides more accurate results, particularly in phase coexistence.^67^ However, mutual magnetization calculations represent a computationally intensive task requiring solution of a many-body problem. Therefore, we focus exclusively on determining the qualitatively optimal lattice configurations in densely packed phases—specifically within the aggregates.

We investigate thermodynamically favorable structures by assuming preset lattice geometries (BCT and Hexagonal), determining the minimum total energy through exploration of different lattice aspect ratios. Results are presented in reduced form as $U^{*}=\frac{\pi \sigma^{3}}{{2\,\;\;\mu }_{0}m^{2}}$ .

## Supplementary Material







## Notes

Influence of surface fraction on the final structure; Averaged dipolar interaction; Magnetic relaxation and time-averaged interaction; Effect of perturbation angle; Influence of surface fraction on the characteristic length; Average cluster size curves of different phases; Long-term evolution of the orthogonal percolating bands; Periodicity of orthogonal percolating patterns; Magnetization curve of the particles (PDF)
Close microscopy of early self-assembly under biaxial toggled fields (Video S1) (MP4)
Internal aggregate dynamics in orthogonal percolating structures at 0.3, 1, and 3 Hz (Video S2) (MP4)
