# Supplementary material for: Template-Free Ultrafast Directed Self-Assembly Using Biaxial Toggled Magnetic Fields
Source: ACS Nano. 2025 Jul 30;19(31):28873–87. doi: 10.1021/acsnano.5c09450 (PMC12356125; doi:10.1021/acsnano.5c09450)
Supplement: Supplementary file 1 [file nn5c09450_si_001.pdf]

Supplementary Information for:

Template-free Ultra-fast Directed Self-assembly using Biaxial Toggled Magnetic Fields

*Guillermo Camacho and Juan de Vicente**

F2N2Lab, Magnetic Soft Matter Group, Department of Applied Physics, Faculty of Sciences, University of Granada, C/Fuentenueva s/n, 18071 Granada, Spain

*jvicente@ugr.es

S1 – Influence of surface fraction on the final structure

The morphology of the final structures can depend on particle surface fraction, affecting the way self-assembly proceeds. To test this hypothesis, different concentrations were explored, from $\phi_{2D} = 0.20$ to $\phi_{2D} = 0.90$. To this end, we fixed the main field strength to $H_y = 1.5$ kA·m^{-1} and the frequency to $f = 1$ Hz and swept a range of perturbation strengths from $H_x/H_y = 0.05$ to $H_x/H_y = 1$. Results are presented in Figure S1.1.

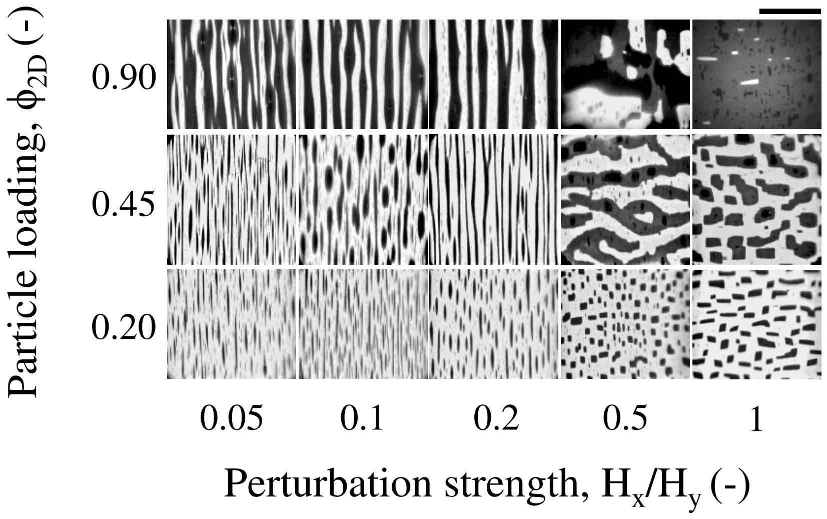

Figure S1.1. Micrographs of the structures formed after applying a sequence of BTFs for 3000 s as a function of particle surface fraction ϕ_{2D} and perturbation strength H_x/H_y. Primary field strength is fixed to $H_y = 1.5$ kAm^{-1} and the field frequency is fixed to $f = 1$ Hz. The black scale bar in the top right corner indicates 500 μm.

At low surface fractions, $\phi_{2D} = 0.2$, percolation of the structures is no longer achievable and the size of the structures for a given field configuration is smaller. Conversely, at high volume fractions, $\phi_{2D} = 0.9$, we face opposite behavior; we are not able to observe any depercolated structures. Interestingly, although columnar structures are not able to depercolate and condense into ellipsoidal aggregates, we observe the Rayleigh-Plateau instability at weak perturbations, as seen by the undulated profile of the particle columns. It has already been shown how depercolation in uniaxial toggled fields (UTFs) proceeds via this instability [1].

S2 – Averaged dipolar interaction

Figure S2.1 shows vector plots of the averaged interaction field for different perturbation strengths, highlighting both radial and rotational components. As the perturbation strength increases, the lateral repulsion vanishes, promoting the coalescence of particles into bands. Plots are scaled to the force prefactors and distance decay for better visualization.

$$\vec{f}_{BTF} = -\frac{3\mu_c m_y^2}{4\pi r^4}\left\{\left[(3\cos^2\theta - 1) + \left(\frac{H_x}{H_y}\right)^2 (3\sin^2\theta - 1)\right]\hat{r} + \left[1 - \left(\frac{H_x}{H_y}\right)^2\right]\sin(2\theta)\,\hat{\theta}\right\}$$

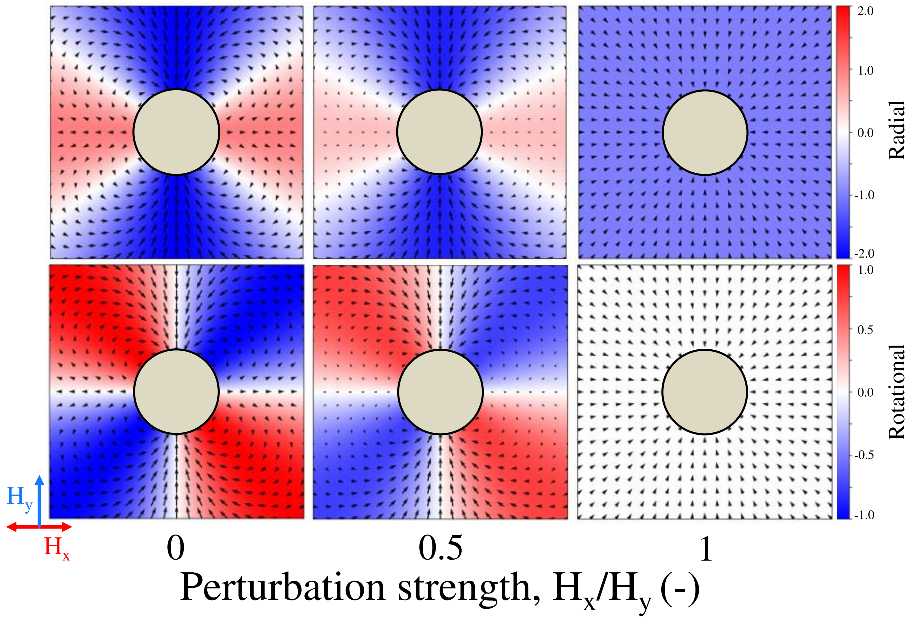

Figure S2.1. Cycle-averaged interaction force field $\vec{f}_{BTF}$ between two dipoles in the XY plane under BTF conditions, shown for different perturbation strengths. The field is scaled as $\frac{4\pi}{3\mu_0 m_y^2} r^4 \vec{f}_{BTF}$ for visualization clarity. Each row displays a vector plot overlaid with a colormap representing, respectively, the radial (top) and rotational (bottom) components of the force. As the perturbation increases, the rotational component progressively vanishes and the interaction becomes increasingly isotropic.

S3 – Magnetic relaxation and time-averaged interaction

At first glance, one might expect isotropic structures when $H_x = H_y$, as the system appears symmetric. However, we observe a preferential alignment of structures along the x-direction, which we attribute to kinetically-driven symmetry breaking induced by magnetic relaxation dynamics during field switching.

In the early stages of aggregation, when clusters are still small and relatively free to move, a slight rotational motion of the aggregates is induced upon field inversion. This originates from finite magnetic relaxation times, which delay the alignment of the dipole moments with the new field direction. As a result, small aggregates undergo a subtle swinging motion around the primary field axis after each switch. This swinging promotes lateral particle migration and favors growth in the direction perpendicular to the primary field. This mechanism is reminiscent of observations by Donado and coworkers [2], who reported similar behavior under combined steady and oscillatory fields.

Although magnetic relaxation in our system is relatively fast, it is sufficient—when combined with periodic field switching and chain breakup—to drive this lateral growth. The outcome is an emergent anisotropy along the perturbation direction, even for $H_x = H_y$, where no directional preference is expected based on field strength alone. This effect is clearly visible in Supplementary Video SV1, which shows the early-stage aggregation under symmetric fields ($H_x = H_y = 2.1$ kA·m^{-1} and $f = 1$ Hz). As shown, aggregates experience slight rotations upon field switching, which tear apart chains along the primary axis and promote lateral growth.

To further demonstrate that this is a kinetic and directional swinging effect, we performed two additional experiments with $H_x = H_y$:

1. Swing-suppressed configuration: We alternated not only the perturbation direction, but also the sign of the primary field component, such that the field vector completes a full rotation per cycle (see Figure S3.1b). In this configuration, the system lacks a preferred swinging axis. The resulting structures after 3000 s are isotropic, supporting the idea that swinging around a fixed axis is key to the observed anisotropy.

2. Fixed-sign configuration: We suppressed all sign inversions during the cycle (i.e., keeping field components always positive). In this case, the average direction of the field corresponds to 45°, and indeed, the structures align along this direction—further confirming that even slight asymmetries in the time-averaged field due to relaxation can dictate the orientation of the resulting structures (see Figure S3.1c).

In summary, while the field configuration may appear symmetric for $H_x = H_y$, non-instantaneous magnetic relaxation introduces temporal asymmetries that break this symmetry dynamically. This gives rise to a preferred direction of aggregation, driven not by energetic minimization but by relaxation kinetics and time-averaged anisotropic interactions.

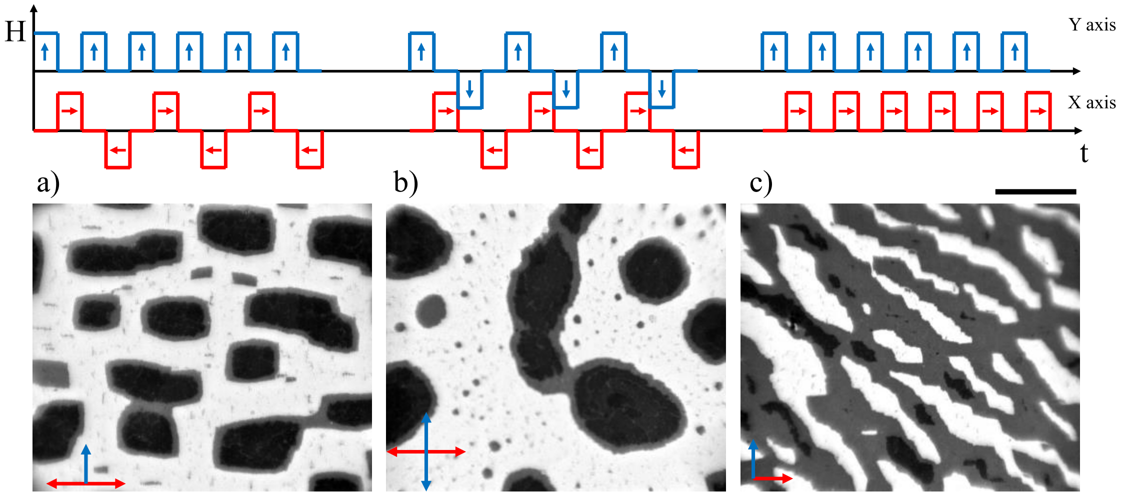

Figure S3.1. Final microstructures formed after 3000 s under different BTF field configurations, illustrating the role of field sign alternation. (a) The perturbation component switches sign at each cycle, while the primary field remains constant (configuration used in this work). (b) Both the primary field and perturbation components switch sign at each cycle, eliminating any preferred swinging axis. (c) No sign inversion is applied. In all cases, $H_x = H_y = 2.1$ kA·m^{-1} and $f = 1$ Hz. The scale bar represents 250 µm.

S4 – Perturbation angle

Apart from orthogonal magnetic fields, we also performed experiments using various perturbation angles and perturbation strengths, analyzing both the resulting mesostructures and microstructures. As shown in Figure S4.1, varying the perturbation angle leads to intermediate structural states between the limiting cases of uniaxial toggled fields (UTFs) and orthogonal toggled fields.

At small perturbation angles, the system displays two limiting behaviors depending on the perturbation strength: weak perturbations recover UTF-like behavior, while strong perturbations resemble steady-field conditions, yielding interconnected chains. Intermediate angles produce mesostructures that percolate in multiple directions rather than forming strictly horizontal or vertical networks. This suggests that the directionality and connectivity of the aggregates can be finely tuned by adjusting the perturbation angle.

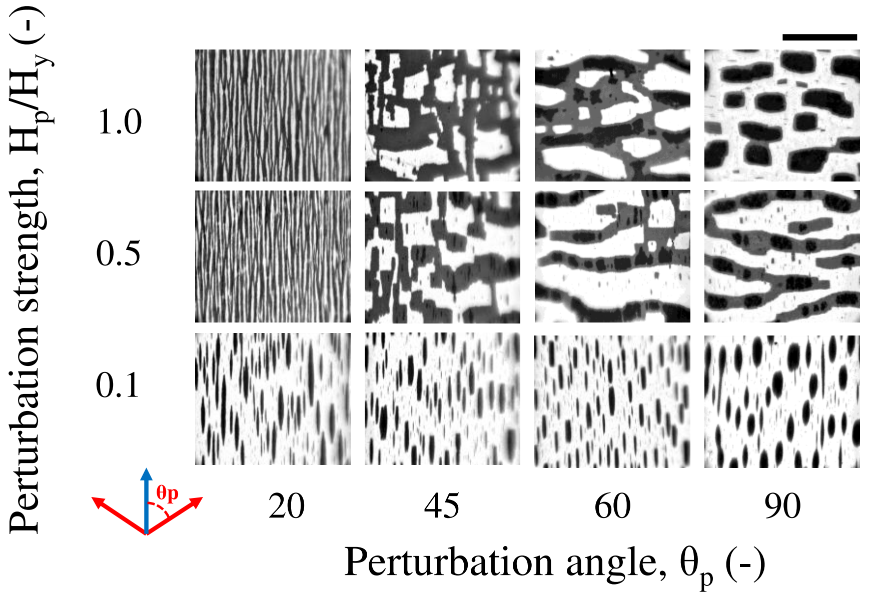

Figure S4.1. Optical micrographs of the structures formed after applying a sequence of BTFs for 3000 s at varying perturbation strengths and angles. The black scale bar in the top right corner corresponds to 500 µm.

Regarding the microstructure, Figure S4.2 compares internal crystalline arrangements under identical field strengths but with perturbation angles of $\theta_p = 90°$ and $60°$, respectively. The main distinction lies in the orientation of the crystalline domains. While

the 90° configuration results in domains with diverse orientations, the 60° configuration tends to produce better-aligned domains, with lattice vectors consistently oriented along the field axes. Taking as a reference the lattice axis closer to main field axis, maximum orientation deviations for the orthogonal perturbation are 35º, while this deviation reduces to only 4.4º for the 60º perturbation. This observation supports the idea that angular control could be used to guide not only mesoscale morphology but also internal order.

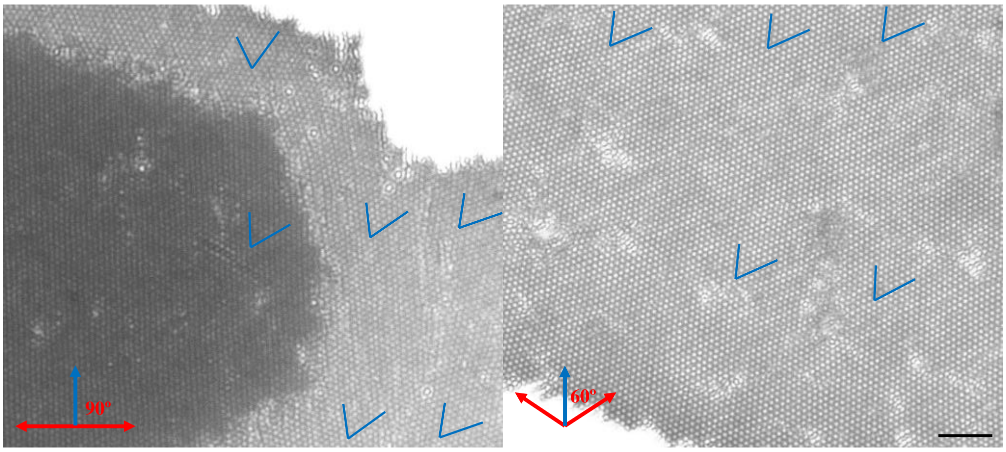

Figure S4.2. Optical micrographs showing the internal crystalline arrangement of structures formed under biaxial toggled fields (BTFs) with different perturbation angles. (a) Fields toggled at 90° (orthogonal configuration). (b) Fields toggled at 60°. Blue lines indicate the orientation of lattice vectors in selected crystalline domains for visual guidance. For both experiments shown $H_y = H_p = 2.1$ kA·m^{-1} and $f = 1$ Hz. Scale bar represents 10 µm.

S5 – Influence of surface fraction on the characteristic length

Despite morphological differences in the final structures across volume fractions (see Section S1), the dynamic evolution of the structures remains consistent across concentrations. For this reason, the manuscript focuses on results obtained at a single particle loading ($\phi_{2D} = 0.45$). The characteristic length L_{char} continues to collapse onto a single master curve when rescaled by the aggregation time $t^* = t/t_{a,x}$, where:

$$t_{a,x} = \frac{2\eta}{5\mu_0\beta^2 H_x^2}\left(\frac{\pi}{6\phi_{2D}}\right)^{\frac{5}{2}}$$

As shown in Figure S5.1, the primary effect of particle loading is a shift in the aggregation timescale, with minimal impact on the overall scaling behavior. However, due to the finite field of view in our imaging setup, L_{char} cannot capture large-scale divergence for horizontally percolating structures, which are expected to exceed the resolution limit of our technique. Additionally, for low surface fractions and weak perturbations, the assembly process becomes dominated by Brownian motion and thus does not conform to the same scaling. This is consistent with previous studies showing a different dependence on particle loading in the Brownian regime ($t_B \propto \phi_{2D}^{-1}$) [3, 4].

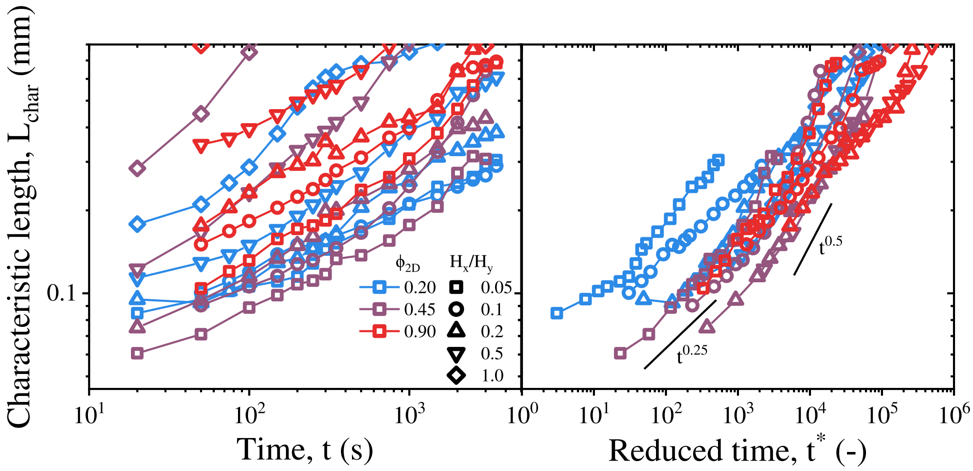

Figure S5.1. a) Temporal evolution of the characteristic length L_{char} of the assembled structures for different volume fractions ϕ_{2D} and perturbation strengths H_x/H_y. b) Master curve showing the collapse of L_{char} when plotted against the reduced time $t^* = t/t_{a,x}$, highlighting the universal scaling behavior across concentrations.

S6 – Average cluster size curves of different phases

The curves shown in Figure 8 were carefully selected to represent the characteristic behaviors observed across all experimental conditions explored in this work. After systematically analyzing the full set of experimental data, we concluded that each phase in the diagram exhibits either a unique kinetic behavior or two distinct ones, as described in the manuscript. We therefore limited the number of curves in the figure to one or two per phase to avoid redundancy and maintain visual clarity.

To further support the representativeness of the selected curves, we now include additional kinetic data in Figure S6.1, showing a wide range of $S(t)$ curves across different field strengths and parameter combinations. These plots confirm that no qualitatively different kinetic regimes were observed beyond those already presented in Figure 8. For percolating structures, a single kinetic behavior was consistently found. In the depercolated regime, two types of dynamics appear. No evidence of continuous variation of the power-law exponents with field parameters was found; slight variations in slope are attributed to experimental dispersion rather than to systematic effects of the magnetic fields.

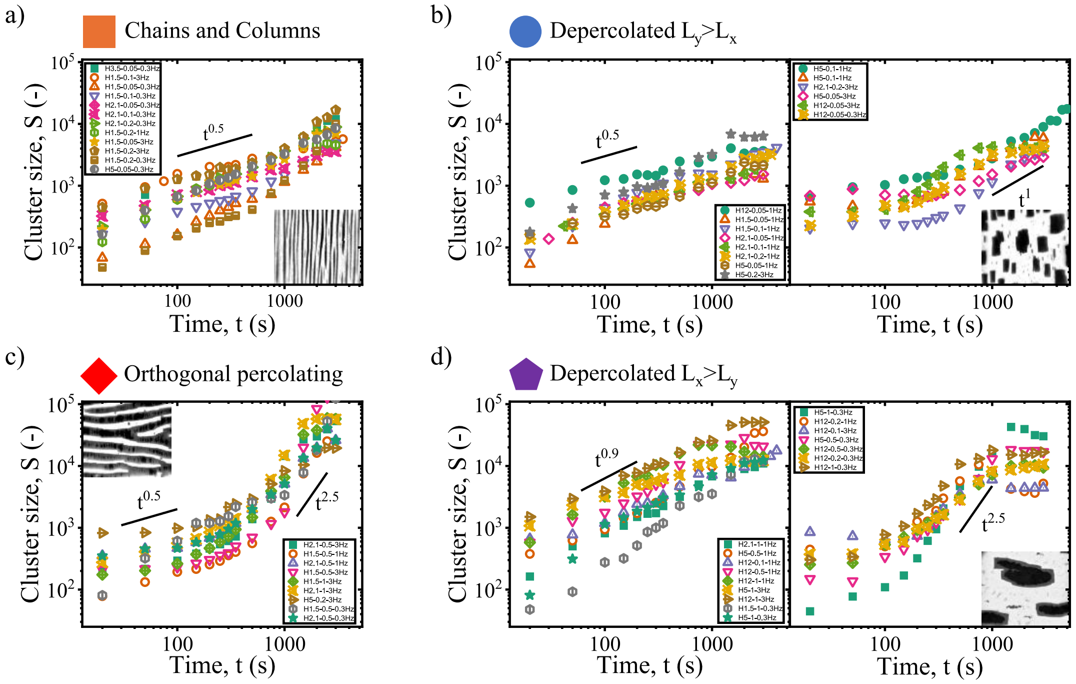

Figure S6.1. Mean cluster size $S(t)$ for multiple experiments across all observed structural regimes. Each subplot corresponds to one of the structural phases discussed in the manuscript. Within each, we show

results from several experiments performed under different field conditions to demonstrate the robustness and generality of the observed kinetic behaviors. The legend indicates experimental parameters using three values: main field strength (in $kA \cdot m^{-1}$), relative perturbation strength H_x/H_y, and toggling frequency (in Hz), respectively.

S7 – Long-term evolution of the orthogonal percolating bands

Experiments reveal that in BTFs, the most stable configuration under the studied conditions consists of flat, horizontal bands orthogonal to the primary field direction. This is evidenced by extended-time experiments (up to 8500 s) conducted at $H_y = 2.1$ kA·m^{-1}, $H_x/H_y = 0.5$, $f = 1$ Hz, presented in Figure S7.1. These results show that while the system reaches horizontal percolation around 3000 s, morphological evolution continues more slowly beyond this point; wavy interfaces progressively transition into flat, horizontal edges through local particle rearrangements along the aggregate surfaces. This flattening mechanism, rather than further growth or coarsening, dominates the post-percolation dynamics.

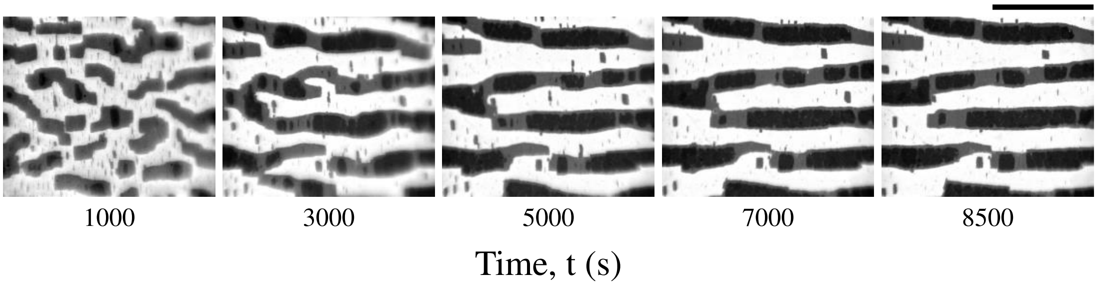

Figure S7.1. Time-lapse micrographs showing the structural evolution of orthogonal bands during a long-duration experiment under band formation conditions. $H_y = 2.1$ kA·m^{-1}, $H_x/H_y = 0.5$, $f = 1$Hz. The scale bar corresponds to 500 μm.

S8 – Periodicity of orthogonal percolating patterns

Vertical coarsening ceases once percolation is achieved, and the aggregate size remains essentially constant thereafter. Therefore, the structures do not continue to grow indefinitely, but rather evolve toward a stable shape characterized by minimized interface curvature and horizontal alignment.

The limited extent of the band formation region in the phase diagram restricts the number of observable bands, which precludes the use of Fourier-based power spectrum methods employed elsewhere in the manuscript. Instead, we can estimate the vertical characteristic spacing $L_{char,y}$ by counting the number of bands crossing vertical lines in the micrographs, using $L_{char,y} = L_{total}/N_{bands}$ where L_{total} is the vertical field of view. From the data shown in Figure 2, we find that $L_{char,y}$ increases systematically with increasing H_y (see Figure S8.1). This relationship holds across different frequencies and exhibits a linear trend with H_y, consistent with previous studies in rotatory fields at high particle loading, where isotropic percolating structures were reported [5].

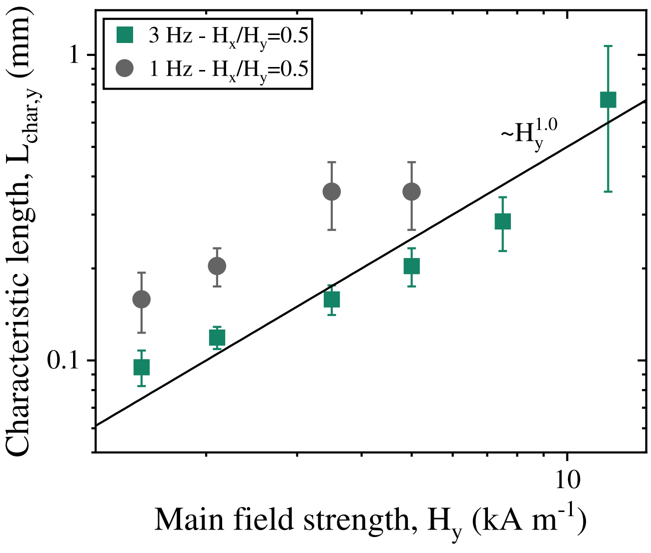

Figure S8.1. Characteristic length scale of orthogonal bands along the primary field direction $L_{char,y}$ as a function of the applied field strength H_y, for two excitation frequencies (f = 1 and 3 Hz). Data for f = 0.3 Hz are not included, as the formation of well-defined orthogonal structures was insufficient under these conditions to allow for reliable measurement.

S9 – Magnetization curve of the particles

The particles used in this work are magnetically soft. Figure S9.1 presents the magnetization curve of the particle powder, measured using a Quantum Design MPMS XL magnetometer. As shown, there is no detectable remanent magnetization. The inset highlights the low-field region around $H = 0$ kA·m^{-1}, confirming a linear magnetization response up to $H \sim 12$ kA·m^{-1}, which corresponds to the maximum field strength used in this study. The initial magnetic susceptibility is $\chi = 1.4$.

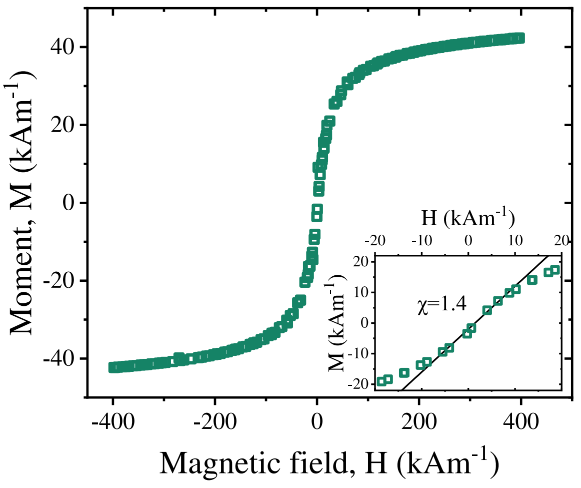

Figure S9.1. Room-temperature magnetization curve of Dynabeads MyOne Carboxylic Acid particles measured using a Quantum Design MPMS XL magnetometer. The inset zooms the linear magnetization regime.

S10 – Supplementary Videos

SV1. Early stage self-assembly of a sample under Biaxial Toggled Fields with $H_y = H_x = 2.1$ kA·m^{-1} and $f = 1$ Hz. Magnetic relaxation induces oscillatory motion of chains and aggregates around the main field axis. Videomicroscopy was performed using a Leica DMI3000 B microscope with 100× magnification.

SV2. Internal aggregate dynamics in orthogonal percolating structures at three different frequencies: 0.3 Hz (left), 1 Hz (center), and 3 Hz (right). The applied field conditions were $H_y = 2.1$ kA·m^{-1} and $H_x/H_y = 0.5$. Chain rotation and net particle migration along the edges of aggregates are observed. Videomicroscopy was performed using a Leica DMI3000 B microscope with 100× magnification.

References

[1] Bauer, J. L., Liu, Y., Kurian, M. J., Swan, J. W., & Furst, E. M. (2015). Coarsening mechanics of a colloidal suspension in toggled fields. Journal of Chemical Physics, 143(7). https://doi.org/10.1063/1.4927563

[2] Donado, F., Sandoval, U., & Carrillo, J. L. (2009). Kinetics of aggregation in non-Brownian magnetic particle dispersions in the presence of perturbations. Phys. Rev. E 79, 011406. https://doi.org/10.1103/PhysRevE.79.011406

[3] Promislow, J. H. E., Gast, A. P., & Fermigier, M. (1995). Aggregation kinetics of paramagnetic colloidal particles. J. Chem. Phys. 102, 5492–5498. https://doi.org/10.1063/1.469278

[4] Shahrivar, K., Carreón-González, E., Morillas, J. R., & de Vicente, J. (2017). Aggregation kinetics of carbonyl iron based magnetic suspensions in 2D. Soft Matter, 13, 2677. https://doi.org/10.1039/C7SM00075H

[5] Hilou, E., Joshi, K. & Biswal, S. L. (2020). Characterizing the spatiotemporal evolution of paramagnetic colloids in time-varying magnetic fields with Minkowski functionals. Soft Matter, 16, 8799. https://doi.org/10.1039/D0SM01100B
